# Identification of different respiratory viruses, after a cell culture step, by matrix assisted laser desorption/ionization time of flight mass spectrometry (MALDI-TOF MS)

**DOI:** 10.1038/srep36082

**Published:** 2016-10-27

**Authors:** Adriana Calderaro, Maria Cristina Arcangeletti, Isabella Rodighiero, Mirko Buttrini, Sara Montecchini, Rosita Vasile Simone, Maria Cristina Medici, Carlo Chezzi, Flora De Conto

**Affiliations:** 1 Department of Clinical and Experimental Medicine – Unit of Microbiology and Virology - University of Parma – Parma, Italy

## Abstract

In this study matrix assisted laser desorption/ionization time of flight mass spectrometry (MALDI-TOF MS), a reliable identification method for the diagnosis of bacterial and fungal infections, is presented as an innovative tool to investigate the protein profile of cell cultures infected by the most common viruses causing respiratory tract infections in humans. MALDI-TOF MS was applied to the identification of influenza A and B viruses, adenovirus C species, parainfluenza virus types 1, 2 and 3, respiratory syncytial virus, echovirus, cytomegalovirus and metapneumovirus. In this study MALDI-TOF MS was proposed as a model to be applied to the identification of cultivable respiratory viruses using cell culture as a viral proteins enrichment method to the proteome profiling of virus infected and uninfected cell cultures. The reference virus strains and 58 viruses identified from respiratory samples of subjects with respiratory diseases positive for one of the above mentioned viral agents by cell culture were used for the *in vitro* infection of suitable cell cultures. The isolated viral particles, concentrated by ultracentrifugation, were used for subsequent protein extraction and their spectra profiles were generated by MALDI-TOF MS analysis. The newly created library allowed us to discriminate between uninfected and respiratory virus infected cell cultures.

Rapid identification of respiratory viruses is a major goal in starting early therapy and prophylaxis and preventing outbreaks or epidemics^1^,^2^,^3^,^4^. Currently, nucleic acid detection, serology, cell culture and, in some cases, electron microscopy are used in virological diagnosis^1^,^2^,^3^,^4^. However, serology and polymerase chain reaction (PCR) based assays are specific target directed and thus could miss non-selected viruses. On the other hand, although not target directed and then potentially able to detect all the cultivable viruses, cell culture is time consuming, expensive and needs accessory assays and trained personnel for virus identification, such as viral antigens detection by enzyme immunoassays, immunofluorescence^5^,^6^,^7^,^8^ or electron microscopy.

The rapid detection of viral agents causing respiratory diseases is a key for an effective infection control^7^. It is therefore understandable the growing interest in developing novel assays or technology rapid and easy to use in routine diagnostic laboratory^7^,^9^.

Matrix-assisted laser desorption/ionization time of flight mass spectrometry (MALDI-TOF MS) is a rapid and sensitive technology in clinical microbiology, and economical also in terms of both workload and costs^10^,^11^,^12^,^13^,^14^,^15^.

Despite many literature data about MALDI-TOF MS application in routine clinical microbiology, and in experimental approaches related to bacteria, parasites, yeasts and moulds^6^,^16^,^17^,^18^,^19^,^20^,^21^,^22^, very few reports are available about its application in virus research and laboratory diagnosis of viral infections^6^,^23^,^24^,^25^. In most of the studies, specific viral genome sequences are previously amplified by PCR and then the amplicons are analysed by MALDI-TOF MS^15^.

The aim of this study was the development of a new tool based on MALDI-TOF MS method as a model to be applied to the identification of cultivable respiratory viruses using cell culture step as a viral proteins enrichment method to the proteome profiling of virus infected and uninfected cell cultures.

To this end, a new library was created with the spectra obtained by the American Type Culture Collection (ATCC) and/or United Kingdom National External Quality Assessment Service (UK-Neqas) reference viruses after a cell culture step. The new library was evaluated using cell cultures infected with viruses isolated from respiratory samples collected and analysed by routine diagnostic methods at the Unit of Virology of the University Hospital of Parma (Italy), and whose reference spectra are present in the created library.

## Results

### MALDI-TOF MS analysis of respiratory virus reference strains infected cells

In order to validate the cell culture step as a method of protein enrichment as reported in the “Methods” section, we first analysed the spectra of reference viruses infected cell cultures (influenza A and B viruses, adenovirus C species types 2 and 5, parainfluenza virus types 1, 2, and 3, respiratory syncytial virus, echovirus type 30, and cytomegalovirus) as well as those of cells infected by a metapneumovirus clinical isolate used in this study as reference strain.

The spectra obtained from MDCK SIAT1, LLC-MK2, MRC5, and Intestine 407 uninfected cell lines were used as a baseline for the detection of any differences in comparison with the spectra derived from cell cultures infected with the tested viruses.

**Figure 1:**
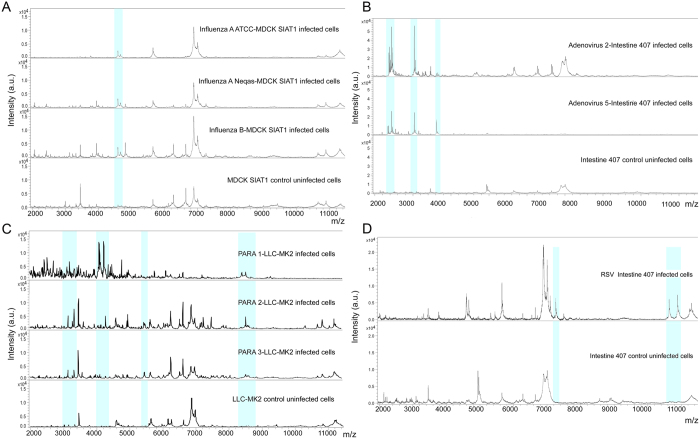
Representative MALDI-TOF mass spectra showing peaks in Linear Positive mode acquired in MBT-FC method analysed in the range 2–20 KDa. (**A**) Detail of the spectra of ATCC and Neqas 1217 influenza A infected cell cultures (“Influenza A ATCC-MDCK SIAT1 infected cells” and “Influenza A Neqas-MDCK SIAT1 infected cells”) and Neqas 1785 Influenza B infected cell cultures (“Influenza B-MDCK SIAT1 infected cells”), compared with the spectrum of uninfected cell cultures (“MDCK SIAT1 control uninfected cells”). (**B**) Detail of the spectra of adenovirus type 2 and 5 reference strains both cultured in Intestine 407 cell line (“Adenovirus 2-Intestine 407 infected cells”, “Adenovirus 5-Intestine 407 infected cells”) compared with the spectrum profile of uninfected control cell cultures (“Intestine 407 control uninfected cells”). (**C**) Detail of the spectra of parainfluenza virus type 1, 2, and 3 cultured in LLC-MK2 cell line *versus* the spectrum profile of uninfected cell cultures (“PARA 1-LLC-MK2 infected cells”, “PARA 2-LLC-MK2 infected cells”, “PARA 3-LLC-MK2 infected cells” and “LLC-MK2 control uninfected cells”, respectively). (**D**) Detail of the spectra of RSV reference strain reproduced in Intestine 407 cell line (“RSV-Intestine 407 infected cells”) compared with “Intestine 407 control uninfected cell”. All spectra were baseline subtracted, smoothed, normalized and realigned. The x-axis shows the m/z value and the y-axis the intensity expressed in arbitrary units (a.u.) of each peak. Light blue rectangles highlight some of the peaks detected by FlexAnalysis software with a 100% frequency in all replicates and reported in Table S1.

**Figure 2:**
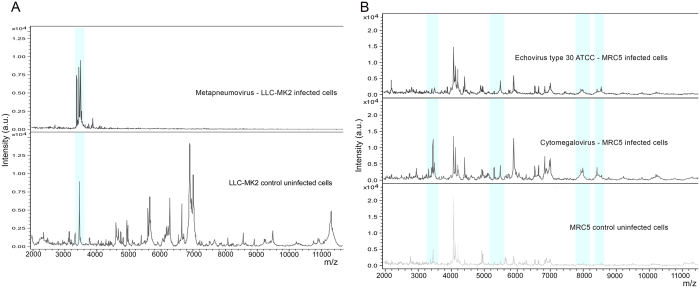
Representative MALDI-TOF mass spectra showing peaks in Linear Positive mode acquired in MBT-FC method analysed in the range 2–20 KDa. (**A**) Detail of the spectra of metapneumovirus cultured in LLC-MK2 cell line (“Metapneumovirus-LLC-MK2 infected cells”) *versus* the spectrum profile of uninfected cell cultures (“LLC-MK2 control uninfected cells”). (**B**) Detail of the spectra of Echovirus type 30 infected cell cultures (“Echovirus type 30 ATCC – MRC5 infected cells”), human cytomegalovirus cultured in MRC5 cell line (“Cytomegalovirus– MRC5 infected cells”), compared with the spectrum of uninfected cell cultures (“MRC5 control uninfected cells”). All spectra were baseline subtracted, smoothed, normalized and realigned. The x-axis shows the m/z value and the y-axis the intensity expressed in arbitrary units (a.u.) of each peak. Light blue rectangles highlight some of the peaks detected by FlexAnalysis software with a 100% frequency in all replicates and reported in Table S1.

The spectra generated from virus infected cell cultures, analysed in the molecular weight range 2000–20,000 Da, revealed, in the molecular weight range 2000–11,200 Da, the presence of some different peaks not overlapping those of uninfected cell cultures for all the reference virus infected cell cultures, with a frequency of 100% in all the replicates (Figs 1 and 2). The parameters for the creation of the Main Spectrum Profile (MSP) for each of the reference virus infected cell cultures were set on the basis of the peaks’ value reported in Table S1, as detailed in the “Methods” section. The MSP for each uninfected cell line was also created. The obtained MSP spectra were used to create a new respiratory viruses library in our Bruker Daltonics database in order to blind identify viruses isolated from biological samples after a cell culture step.

The reference virus infected cell cultures profiles were correctly identified in different independent experiments, performed by different operators in different days on the basis of the newly created respiratory viruses library with an average score value of the first best match >2.0 for each spectra set obtained for each data point (different cultures of different operators in different days); the statistical analysis of the results obtained were showed in supplementary Figure S2. However, in some replicates discrimination between influenza A and B was not obtained. These latter spectra match with both MSP of influenza A and B (first or second best match, alternatively) with a score value >2.0.

**Figure 3:**
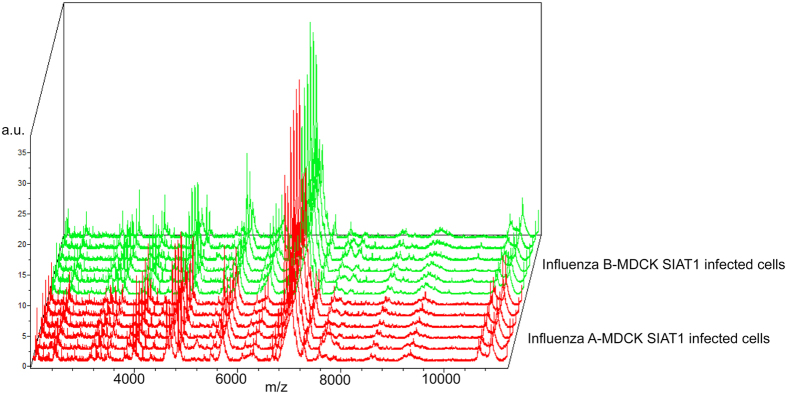
Spectra replicates of human influenza A virus reference strain (“Influenza A-MDCK SIAT1 infected cells”, red spectra) and of the influenza B reference strain (“Influenza B-MDCK SIAT1 infected cells”, green spectra) analysed by ClinProTools Software. The x-axis records the m/z value, the y-axis the peaks intensity expressed in arbitrary units (a.u.) and the z-axis the loading order.

Analysis of the spectra with a software dedicated to discover biomarkers (ClinProTools) revealed the absence of discriminating peaks for the differentiation between influenza A and B infected cell cultures (Fig. 3) in the mass range considered. No misidentification with MSP of uninfected cell lines was found.

**Figure 4:**
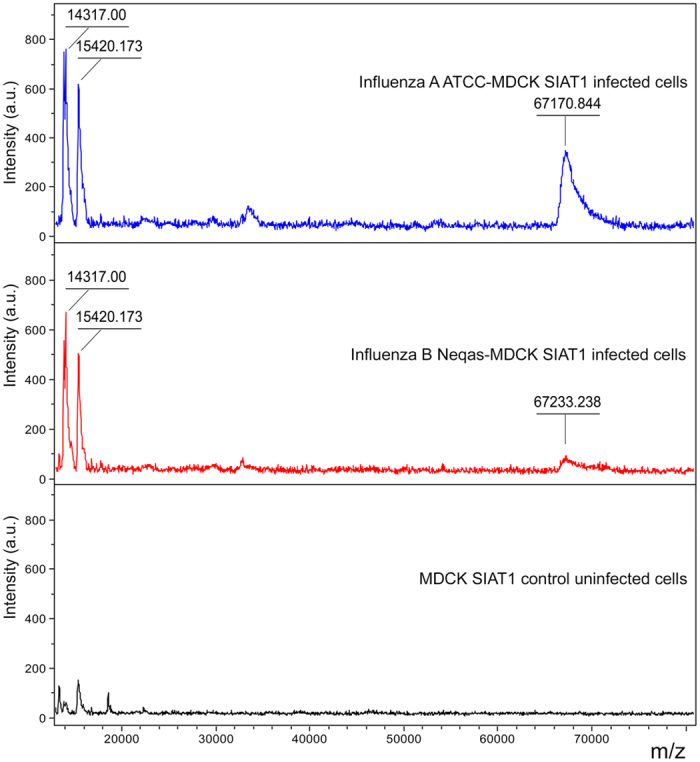
Representative MALDI-TOF mass spectra of influenza A and B infected cell cultures (“Influenza A-MDCK SIAT1 infected cells” and “Influenza B-MDCK SIAT1 infected cells”) showing peaks in Linear Positive mode acquired in high molecular weight, with pointed average masses, compared with the spectra of uninfected cell cultures (“MDCK SIAT1 control uninfected cells”) in the m/z range of 13–80 KDa. The x-axis records the m/z value and the y-axis the intensity expressed in arbitrary units (a.u.) of each peak. All spectra were baseline subtracted, smoothed, normalized and realigned. Molecular weight values of virus and cellular discriminant peaks are indicated on peak tops.

In order to deepen the misidentification between influenza A and B infected cell cultures, the spectra of the reference strains of influenza A and B infected MDCK SIAT1 cells and the corresponding uninfected cell cultures were analysed in a higher molecular weight region (13–80 KDa). The compared spectra highlighted the presence of a peak at 14.3 KDa and a peak at about 67 KDa, which were completely missing in the uninfected cell cultures and a peak at 15.4 KDa present also in uninfected cell cultures (Fig. 4). These peaks were found both in the reference strains of influenza A and B infected MDCK SIAT1 cells.

### Identification of viruses isolated from respiratory tract samples after a cell culture step by the newly created respiratory viruses library

Based on these preliminary results, the spectra of cell cultures infected by respiratory viruses identified after isolation from biological samples were analysed. The MALDI-TOF MS analysis was performed, after a cell culture step, on 58 isolates representative of respiratory tract viruses, derived from different biological samples (nasopharyngeal aspirates, throat swabs, bronchoalveolar aspirates, bronchoalveolar lavages, and an orotracheal aspirate), identified by routine diagnostic methods including cell cultures and in the majority of cases also with real-time PCR assays and stored at −80 °C.

The protein spectra of respiratory syncytial virus (RSV), echovirus type 30, cytomegalovirus and metapneumovirus infected cell cultures were correctly identified with score values >2.2 for all the replicates tested when compared with the newly created respiratory viruses database by using the MALDI Biotyper software. The protein spectra of influenza A and B virus infected cell cultures were all identified as human influenza virus with a score value ≥ 2.0 for all the replicates without discrimination between A and B species, as also reported for the corresponding reference strains. The protein spectra of parainfluenza virus types 1, 2 and 3 infected cell cultures were all identified as parainfluenza virus with a score value > 2.1 for all the replicates without discrimination between the different types. Concerning the adenoviruses isolated from 8 clinical samples after cell culture, they were identified as adenovirus C species with a score value > 2.0 without discrimination between type 2 and 5. Results by Bruker Daltonics MALDI Biotyper of respiratory viruses isolated from biological samples after a cell culture step are reported in Table 1.

Moreover, for 15 representative of the 58 viruses infected cell cultures derived from biological samples the identification reproducibility was tested by different operators in different days and the statistical analysis of the results obtained were showed in supplementary Figure S3.

The detection limit of the method was 10^6^ tissue culture infectious dose_50_ (TCID_50_)/μl for the viruses tested.

### Identification of Adenoviruses type 2 and type 5 and Parainfluenza virus types 1, 2 and 3

Spectra analysis by ClinProTools Software of the reference type 2 and 5 adenoviruses infected cell cultures used to create MSP, reveals the presence of five discriminating peaks (Table 2). Three peaks (2383, 2397 and 2369 m/z) were characteristic of adenovirus type 2 infected cell cultures and two at 2341 and 2355 m/z were typical of adenovirus type 5 infected cell cultures.

**Figure 5:**
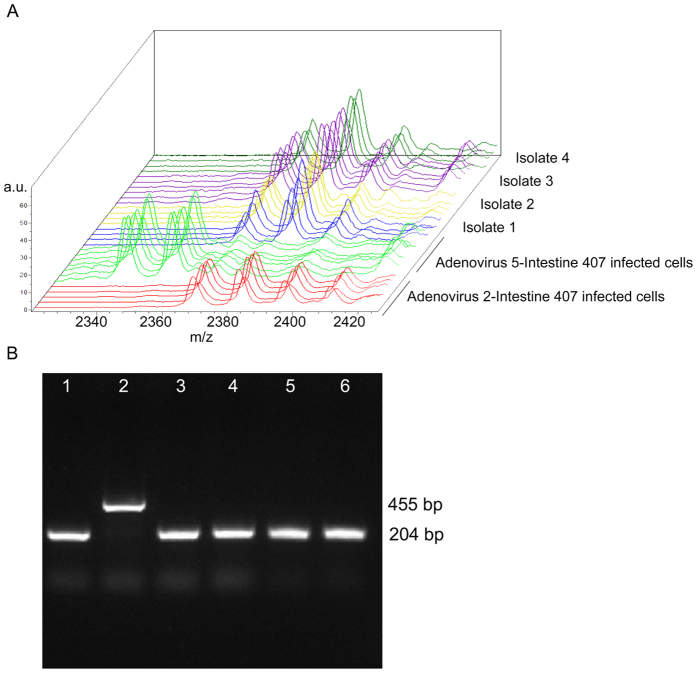
Example of comparative analysis of the adenovirus isolated from biological samples and the reference strains infected cell cultures. (**A**) ClinProTools’ stack view displaying in a three dimensional space the spectra of six model generation classes colored according to their class membership. Reference Adenovirus 2-Intestine 407 infected cell cultures (red spectra), reference Adenovirus 5-Intestine 407 infected cell cultures (green spectra), isolate 1 infected cell culture (blue spectra), isolate 2 infected cell culture (yellow spectra), isolate 3 infected cell culture (violet spectra) and isolate 4 infected cell culture (dark green spectra). The x-axis records the m/z value, the y-axis the peak intensity in arbitrary units (a.u.) and the z-axis the loading order. (**B**) Polymerase chain reaction for the identification of type 2 and type 5 adenoviruses. The reaction products were analysed on 1.5% agarose gels containing ethidium bromide (0.01 mg/ml). Lane 1 reference Adenovirus 2-Intestine 407 infected cells, lane 2 reference Adenovirus 5-Intestine 407 infected cells, lanes 3, 4, 5, 6 isolate 1–4 infected cell cultures, respectively. Expected amplification products molecular weights are indicated on the right of the figure.

Taking into account the peak area/intensity average values, the comparison of cell cultures infected with eight adenoviruses isolated from clinical samples with reference strains infected cell cultures allowed us to identify them as adenovirus type 2 (Table 3, Fig. 5A). This result was confirmed by PCR assay (Fig. 5B).

The same analysis carried out on the spectra of reference parainfluenza virus types 1, 2, 3 infected cell cultures used to create MSP, revealed the presence of 8 discriminating peaks (Table 4): m/z 4084, 4230 for parainfluenza virus type 1; m/z 6275, 6647 for parainfluenza virus type 2; m/z 5655, 6893, 6952, 7006 for parainfluenza virus type 3.

Taking into account the peak area/intensity average values, the comparison of cell cultures infected with all the parainfluenza viruses isolated from clinical samples with the reference strains infected cell cultures allowed us to correctly identify only 6 parainfluenza type 3. For the remaining 5 clinical isolates (2 parainfluenza type 3, 2 parainfluenza type 2 e 1 parainfluenza type 1), the peaks found not allowed to identify any of the three serotypes.

## Discussion

MALDI-TOF MS allows easier and faster identification of human microbial pathogens than conventional identification methods, with unquestionable reliability and cost–effectiveness^12^. The limitation of this technology is that identification of new isolates is possible only if the spectral database contains peptide mass fingerprints of the type strains of specific genera/species/subspecies^15^. Moreover, the MALDI-TOF mass spectrometry is strictly dependent on the analyte concentration in the tested samples. As reported in the literature [i.e. ref. 26], the identification of microorganisms could be directly applied to some clinical samples. However, the major limitation is the amount of analyte present in the samples and the limit of detection of current MALDI-TOF protocols. To circumvent this difficulty, large volumes of samples are used and an additional enrichment by culture is required^26^.

At present, mass spectrometry has had limited application in clinical virology^6^,^23^,^24^,^25^. In this light, the main purpose of this study was the development of a new tool based on MALDI-TOF MS as a model to be applied to the identification of cultivable respiratory viruses after a cell culture step. The present study demonstrated the capability of MALDI-TOF MS to detect specific profiles of cell cultures infected with viral agents causing respiratory tract infections. These new insights could be suitable of further applications of MALDI-TOF MS to clinical and/or basic and applied virology.

The commercially available assays for diagnostic purpose detecting viral nucleic acids (DNA/RNA) usually target adenovirus, parainfluenza virus, respiratory syncytial virus, bocavirus, metapneumovirus, influenza A virus and influenza B virus, as those currently used also in our laboratory. In this study, all these viruses were considered except bocavirus that is known to be particularly difficult to propagate in cell culture^27^. On the other hand the limited number of the parainfluenza virus types 1 and 2 as well as human metapneumovirus strains tested are dependent on the low frequency of these viruses in our setting and, for the latter, also to the difficulty to grow in cell cultures conventionally used for the isolation of respiratory viruses^28^. As a matter of fact in our laboratory in 2014 only parainfluenza virus type 3 was detected by culture, and metapneumovirus had a frequency of 6.8% detected only by real-time PCR (unpublished data). On the contrary, in this study, echovirus type 30 and cytomegalovirus were added having been found them by cultivated clinical samples in a number of respiratory tract infections in our laboratory. MALDI-TOF MS is strictly dependent on the viral concentration in the sample and on the presence of molecules deriving from cell debris^24^. The present study demonstrated that with a minimum concentration of 10^6^ TCID_50_/μl it is able to discriminate between virus infected and uninfected cell cultures. As a matter of fact, the outcomes showed that the spectra profiles of *in vitro* virus infected cell lines had characteristic peaks absent in the corresponding uninfected cell cultures. These peaks might result from the synthesis of viral-specific proteins and/or the effects of the viral infection on the cellular protein synthesis. However, the peptide mass fingerprint by the suitable mass spectrometer could be used for the further identification of these proteins, as the Microflex LT mass spectrometer used in this study does not support such application.

The spectra profiles of both reference influenza A and influenza B virus infected cell cultures were very similar with slight differences in the analysed region (molecular weight 2–20 KDa) not allowing types differentiation.

Although no misidentification between MSP of influenza A and B virus infected MDCK SIAT1 cells and MSP of uninfected cell cultures was found, slight similarities were observed with the protein spectra of this uninfected cell line. The obtained results are unexpected, especially considering that a discrimination among adenovirus C types and parainfluenza types reference strains has been successful by using a discovery biomarkers software (ClinProTools), as also previously observed for poliovirus types^24^. This could be also a consequence of the fact that enveloped viruses have the capability of incorporating some host proteins packaged into their envelope^29^. It is likely that the peaks observed both in infected and in uninfected cell cultures are residual contaminating host proteins and/or host proteins packaged into the virus, considering that proteins were extracted directly from virus particles and residual cell debris obtained by ultracentrifugation.

Interestingly, the comparison of the spectra of reference influenza virus infected MDCK SIAT1 cells with the corresponding uninfected cell cultures, when examined in a higher molecular weight region (13–80 KDa), highlights the presence of peaks absent in the control uninfected cell culture spectra that could be related to specific viral proteins, like the nuclear export protein (NEP, 14,317 Da) and the hemagglutinin (HA, 63,525 Da)^30^. However, the low intensity of these peaks makes them not easy to use as possible biomarkers. Moreover, it should be emphasized that the small difference found in the molecular weight of the proteins at about 67 KDa could not be reliable to discriminate between the two spectra, given the low distance to the limit of detectability of the Microflex LT spectrometer (100 KDa). Closer to this limit, in fact, the instrument shows a gradual loss of resolution.

On the contrary, the reference types 2 and 5 adenovirus infected cell cultures spectra show the presence of discriminating peaks and have few peaks overlapping the corresponding uninfected cell line, probably due to the “life cycle” of the virus^31^. In the case of parainfluenza viruses, the possibility to differentiate the three types was not completely achieved. Although, using ClinProTools software, the differentiation of the types 1, 2 and 3 reference strains was obtained, only 6 parainfluenza type 3 clinical isolates were correctly identified. The low number of clinical isolates tested and the discriminating peaks found for parainfluenza types 1 and 2 (two for each one) could explain the better result obtained for the identification of parainfluenza virus type 3, for which four discriminating peaks were found. For these reasons, the parainfluenza types identification needs to be improved.

Concerning the analysis of the spectra profiles of reference RSV, echovirus type 30, cytomegalovirus, and metapneumovirus, the virus infected cell cultures show peaks profiles not overlapping those of the corresponding uninfected cell cultures.

The newly created database gave interesting results concerning the identification of respiratory viruses isolated from different biological samples from the respiratory tract after a cell culture step, showing a 100% concordance compared with the identification by conventional routine diagnostic methods. The subtypes identification, not allowed by using the in-house database alone, was completely obtained after an accurate statistical analysis of the spectra for all the adenoviruses strains (reference and clinical isolates) and parainfluenza reference strains, and only partially for parainfluenza virus clinical isolates. On the contrary, at present, the discrimination between influenza A and influenza B virus infected cells is not possible because of the absence of differences between the MSP profile of these two viruses.

However, the results of this study demonstrated that changes in the MALDI-TOF MS standard parameters usually used to identify bacteria and fungi allowed to discriminate both between infected and uninfected cell cultures and among the considered respiratory viruses infected cell cultures tested producing the identification of the involved virus. MALDI-TOF MS could present some limitations for its potential application to the diagnostic work-flow of viral respiratory tract infections requiring a high concentration of virus achievable only by culture, but this study could have some importance lying mainly in the development of a modified method for virus identification by this technology as a tool for further application both in clinical and basic/applied virology. However, in those laboratories where the viruses cultivation has been usually performed, MALDI-TOF MS could be applied advantageously, taking into account that the identification subsequent to the culture requires specific fluorescent-labelled monoclonal antibodies targeting the suspected different viruses and experience in the preparation and reading of cell smears^32^. In our laboratory, besides the costs of cultivation (12.16 €), the application of MALDI-TOF MS (0.57 € including the cost of ultracentrifugation) instead of the immunofluorescence (from 3.81 to 15.70 € for each assay depending on the suspected virus) could led to a potential reduction of the costs, excluding the cost of the mass spectrometer.

Moreover, starting from the cytopathic effect observed in viruses infected cell cultures, the main advantages of MALDI-TOF MS, compared with conventional methods for the identification of viruses in cell cultures, are that it is more rapid (about 2 h for MALDI-TOF MS *versus* about 3–9 h for electron microscopy, PCR assays, immunofluorescence or enzyme immunoassays) far less labour intensive, less expensive (excluding the cost of the instrument), not affected by the presence of reaction inhibitors and does not require multiple physically separated areas and specifically trained personnel.

Nevertheless, in those laboratories where only the molecular-based detection and identification are performed, is necessary to take into account that PCR, unlike MALDI-TOF MS, could be applied directly to the biological sample. On the other hand, PCR-based methods, including multiplex assays, are able to detect only known targets. Although the identification of new isolates by MALDI-TOF MS is possible only if the database contains the reference spectrum of the type strain, the model developed in this study is potentially open to all the cultivable viruses, after the implementation of the related reference spectra in the database.

In conclusion, this study could be considered a starting point for further evolutions of the developed system, since the differences observed comparing the virus infected cell culture spectra suggest the possibility to apply this method to the identification of other viruses responsible for respiratory tract infections, as well as to viral agents causing infections of other body sites.

## Methods

### Virus reference strains

The human influenza A virus reference strain 1218 from United Kingdom National External Quality Assessment Service (UK-Neqas), the NWS/33 strain (subtype H1N1) VR219 from American Type Culture Collection (ATCC), and the influenza B (UK-Neqas 7260) were used. The type 2 (UK-Neqas 9054) and type 5 (ATCC; VR-1516) adenovirus C species, the parainfluenza virus type 1 Sendai strain (ATCC; VR-907), the parainfluenza virus type 2 Greer strain (ATCC; VR-92), the parainfluenza virus type 3 (UK-Neqas 1216), the respiratory syncytial virus (UK-Neqas 1217, subtype A2), the human echovirus type 30 (ATCC; VR-322), and the human cytomegalovirus Towne strain (ATCC; VR-977), were also used. Finally, one human metapneumovirus isolated in our laboratory from a nasopharyngeal aspirate and characterized by Rt-PCR assays (Respiratory Multi Well System r-gene^®^ Argene, Italy and Allplex^TM^ respiratory panel 2 – ADV/MPV/HEV/PIV, Seegene, Seoul, Korea) was used as reference strain.

### Viruses isolated from biological samples after cell culture

A total of fifty-eight viruses isolated from as many biological samples collected from the respiratory tract of hospitalized subjects and outpatients with respiratory diseases in different seasons in the period 1993–2016 and analysed at the Virology Unit of the University Hospital of Parma (Italy), were selected and included in this study. In particular, 16 (14 isolated from throat swabs, 1 from a nasopharyngeal aspirate and 1 from a bronchoalveolar aspirate) were positive for human influenza viruses (8 samples positive for influenza A virus and 8 positive for influenza B virus), 8 positive for adenoviruses (2 from nasopharyngeal aspirates and 6 from throat swabs), 1 parainfluenza virus type 1 (from a nasopharyngeal aspirate), 2 positive for parainfluenza virus type 2 (all from nasopharyngeal aspirates), 8 positive for parainfluenza virus type 3 (1 from a bronchoalveolar lavage, 1 from an orotracheal aspirate, 2 from throat swabs and 4 from nasopharyngeal aspirates), 9 positive for respiratory syncytial virus (all from nasopharyngeal aspirates), 8 positive for echovirus type 30 (all from throat swabs), 5 positive for human cytomegalovirus (all from throat swabs), and 1 positive for metapneumovirus (from a nasopharyngeal aspirate).

These samples were previously processed by routine diagnostic methods according to standard procedures including cell cultures and in the majority of cases also with real-time PCR assays (Respiratory Multi Well System r-gene^®^ Argene; Influenza A/B Q-PCR Alert AmpliMIX, Nanogen Advanced Diagnostics S.p.A., Italy) and the viruses isolated by culture were stored at −80 °C until the *in vitro* infection of suitable cell cultures.

The viruses isolated from biological samples analyzed in this study had been obtained by the University Hospital of Parma for routine diagnosis purposes, and no approval by the Institutional Review Board was required because of the laboratory diagnosis results were reported in the medical records of the patients as a diagnostic answer to a clinical suspicion of respiratory tract infection. Ethical approval at the University Hospital of Parma is required only in cases where the biological samples are to be used for applications other than diagnosis. At the University Hospital of Parma informed consent procedures for the laboratory diagnosis of infectious diseases other than HIV serology do not require local committee approval because they are included in the Italian Public Health Legislation. In order to document the process, physicians must write a medical order with the personal and clinical data of each patient who asks for health assistance in a public hospital including the University Hospital of Parma, without any interaction with the patients by the Authors.

### PCR for the identification of type 2 and type 5 adenovirus

Type-specific primers of type 2 and type 5 adenovirus C species were used. The forward primer (5′-TGC TTG CGC THA AAA TGG GCA-3′) was common to all the serotypes of C species, and the reverse primers (5′-CGC TAA GAG CGC CGC TAG TA-3′; 5′-ATG CAA AGG AGC CCC GTA C-3′) were type specific (type 2 and type 5 adenovirus, respectively)^32^. PCR amplification [ref. 33 with minor modifications] was carried out in a 50 μl reaction mixture containing 1 μl aliquots of DNA, extracted using the commercial QIAamp Blood Mini Kit (Qiagen, Italy) according to the manufacturer’s instructions, 5 μl of 10-fold-concentrated buffer, 0.5 μM of each primer, 200 μM of each deoxynucleoside triphosphate, and 1.25 U of *Taq* polymerase (all reagents were from Euroclone, Italy). Thermal cycling consisted of a preliminary denaturation for 3 minutes at 94 °C, 35 cycles of denaturation at 94 °C for 1 minute, annealing at 47 °C for 1 minute, extension at 72 °C for 2 minutes, and a final extension at 72 °C for 7 minutes. The reaction products were analysed on 1.5% agarose gel containing ethidium bromide (0.01 mg/ml) including a 1 Kb molecular mass ladder (Life Technologies Europe, Italy). All products were visualized using a Gel Doc XR instrument (Bio-Rad Laboratories, Italy).

### Cell cultures

Madin-Darby canine kidney cell line permanently transfected by cDNA of the human α-2,6 sialyltransferase (MDCK SIAT1), to express increased levels of α-2,6-linked sialic acid receptors^34^ (kindly provided by Prof. Giovanni Fadda, Institute of Microbiology, Catholic University of the Sacred Hearth, Rome, Italy), were propagated in different lots of Dulbecco Minimum Essential Medium (D-MEM) supplemented with 2 mM L-glutamine, antibiotics (100 U/ml penicillin, 100 mg/ml streptomycin, 2% geneticin G418) and 1% or 10% fetal bovine serum (FBS). Monolayer cultures of human embryonic intestinal cell line (Intestine 407), rhesus monkey kidney epithelial cell line (LLC-MK2), (“Istituto Zooprofilattico Sperimentale della Lombardia e dell’Emilia-Romagna”, Brescia, Italy), and human embryo lung fibroblasts cell line (MRC5) (ATCC) were propagated in different lots of Earle’s modified Minimum Essential Medium (E-MEM), supplemented with 2 mM L-glutamine, antibiotics (100 U/ml penicillin, 100 μg/ml streptomycin), and 1% or 10% FBS^24^. For MRC5 culture, 1% non-essential aminoacids and 1 mM sodium pyruvate were added. All reagents were from Euroclone (Italy).

### Virus infection of cell cultures

For influenza viruses, respiratory syncytial virus, parainfluenza viruses and metapneumovirus infection the cell culture medium was supplemented with trypsin treated with L-1-tosylamide-2-phenylethyl chloromethyl ketone (TPCK, 2 μg/ml), and albumin from bovine serum instead of FBS was added (final concentration 0.1%). For MDCK SIAT1, the cell monolayers grown on 24-well plates were maintained at 37 °C before infection and at 32 °C after infection.

Confluent Intestine 407, LLC-MK2, MRC5, grown for 48 hours on 24-well plates, were inoculated with an aliquot of the above mentioned viruses (previously cultured and frozen at −80 °C). After one hour absorption, the viral inoculum was removed and replaced with a maintenance medium (E-MEM without serum) and infected cells were incubated at 37 °C.

Infected and uninfected control cells and the respective culture media were harvested till a cytopathic effect was clearly observable and then collected in 15 ml conical sterile centrifuge tubes. Collected virus infected and uninfected cells and the respective culture media were stored at −80 °C until further use.

### Virus concentration

After freezing and thawing and cell debris removing by centrifugation at 2000× *g* for 15 minutes (3153 rpm, Centrifuge 5804 R, Eppendorf, Italy), the virus particles and residual cell debris from infected cells, obtained as described above, were immediately ultracentrifuged at 154,300× *g* for 60 minutes at 4 °C (25,000 rpm; SW41 Ti Rotor Optima™ XPN, Beckman Coulter, Italy) and then the pellet subjected to protein extraction for the subsequent analysis by MALDI-TOF MS. The same procedure was applied to uninfected control cells.

The concentration of the virus reference strains tested was performed in duplicate: 1 aliquot (ultracentrifuged pellet suspended in 40 μl of a 1:1 70% formic acid and 100% acetonitrile solution, as described below) was submitted to MALDI-TOF MS analysis and 1 aliquot (ultracentrifuged pellet suspended in 400 μl of medium) to the determination of viral titre by TCID_50_ assay. The aliquot submitted to MALDI-TOF MS analysis was then serially 10-fold diluted in order to evaluate the detection limit of the system related to the viral titre obtained.

### TCID_50_ assay

Serial 10-fold dilutions of the mentioned preparation were used to determine the viral titre expressed as log_10_ of TCID_50_ values per 1000 μl, as previously described^35^.

### Protein extraction and MALDI-TOF MS analysis

The proteins were extracted by using a short extraction protocol as previously described^24^. Briefly, the pellet derived from the ultracentrifugation was resuspended in 20 μl of 70% formic acid (HCOOH) by vigorous mixing followed by the addition of 20 μl of 100% acetonitrile (CAN) (Sigma-Aldrich, Italy) and further vigorous mixing. This mixture was subjected to a second centrifugation step at 16,100× *g* for 2 minutes (13,200 rpm; Centrifuge 5415R, Eppendorf, Italy). For the MALDI-TOF MS analysis 1 μl of the supernatant was spotted onto a MSP 96 polished steel target plate (Bruker Daltonics, Germany), air-dried at room temperature, and overlaid with 1 μl of matrix solution (alpha-cyano-4-hydroxycinnamic acid [4HCCA], diluted in 50% CAN and 2.5% trifluoroacetic acid [TFA], Sigma-Aldrich, Italy) followed by air-drying. MALDI-TOF MS analysis was performed in a 2–20 KDa molecular weight range by using a MicroFlex LT mass spectrometer (Bruker Daltonics, Germany supplied by Becton Dickinson, Italy) instrument.

For the analysis in the high molecular weight range (13–80 KDa) a saturated solution of synapinic acid (SA) was used as the matrix (50 mg/ml SA in 30:70 CAN:TFA 0.1%). Equal volumes (2 μl) of supernatant and matrix solution were mixed. One microliter of this solution was spotted onto the target plate and air-dried at room temperature.

The cell cultures, the protein extraction, the MALDI-TOF MS acquisition, as well as the MALDI-TOF MS analysis were performed by different operators in different days. The data obtained were analyzed by box-and-whisker plot (non-parametric statistics) performed using Excel software.

### MALDI-TOF MS instrumental settings

All the spectra were manually acquired in a positive linear mode by using a Microflex LT mass spectrometer and the FlexControl software (version 3.3.108) (Bruker Daltonics, Germany).

For the acquisition of the spectra the instrument parameters were set on the MBT_FC method of the FlexControl software (positive linear mode; laser frequency 60 Hz, 2–20 KDa molecular weight range). To improve the quality of the spectra, each mass spectrum was generated from the data deriving from several single laser shots in 40-shot steps from different positions of the sample to generate the spectra with an intensity ≥ 10^4^ arbitrary units. To optimize the experimental conditions, a solution of Bruker Bacterial Test Standard (BTS, Bruker Daltonics, Germany) (2–20 KDa range) was used to calibrate the system, according to the manufacturer’s instructions.

For the analysis of larger proteins, in the range of 13–80 KDa, the LP-44 KDa modified method of the FlexControl software was used. In this case, several single laser shots in 100-shot steps from different positions of the sample were acquired to generate the spectra with an intensity ≥ 10^3^ arbitrary units. To optimize the experimental conditions, a solution of Protein Standard II (Bruker Daltonics, Germany) (13–80 KDa range) was used to calibrate the system, according to the manufacturer’s instructions.

Calibration was successful when the protein peaks of the standard were in the range of +/−300 parts per million (ppm) as compared to the respective expected molecular weight.

### MALDI-TOF MS in-house viral database

The raw spectra acquired by MALDI-TOF MS from reference viral strains cultured in different cell lines, as well as from the respective uninfected cell lines, were analysed by FlexAnalysis software to carry out “Smoothing” and “Baseline” and to select homogeneous spectra. For each selected spectrum a peak list including the mass values (m/z), signal-to-noise ratio (S/N), intensity and area, by using MBT_Standard.FAMS Method was created. Subsequently for each viral strain only the peaks present in all replicates (frequency 100%) with a difference of +/−2 Da were selected and the average values of all peak’s parameters calculated. In order to create the in-house viral database, these values were used to set the parameters for the creation of the specific reference Main Spectrum Profile (MSP). The spectra for each viral strain were uploaded in MALDI BioTyper software (version 3.1.66, Bruker Daltonics). The MSP spectrum was calculated in the range 2–11.2 KDa, considering 35 main peaks, by the automated function of BioTyper software (BioTyper MSP Creation Standard Methods) following the manufacturer’s suggestions as previously described^20^,^21^. The “Preprocessing”, “MSP Creation” and “Subtyping MSP Creation” parameters were set as detailed below.

#### Preprocessing

Start (Lower Bound) and end (Upper Bound) of the m/z range for peak peaking were 2–11.2 KDa, respectively. Minimum distance between two peaks along the x-axis which is necessary to separate the peaks from each other (resolution) was 1 Da. The maximum number of peaks to pick (max. peaks) was 35. The peaks must have an intensity of at least 1% of the highest peak (threshold: 0.01). Signal-to-noise ratio (S/N) for a peak which has to be reached must be at least 3.

#### MSP Creation

The initial mass error to be given along the whole spectrum for recalibration (max. mass error of each single spectrum) to separate the preprocessed spectra into the defined mass range was 1000 ppm. The final maximal mass error (desired mass error for the MSP) for each peak after recalibration was 200 ppm. The desired frequency a peak should have within the spectra set (desired peak frequency minimum) to be included in the MSP was 100%. The maximal number of peaks to be included (max. desired peak number for the MSP) was 30. The preprocessing parameters were saved within the MSP by checking the option “generate individual preprocess parameters for MSP”.

#### Subtyping MSP Creation

Lower Bound 2000 Da, Upper Bound 11,200 Da, Max. peaks 25 and threshold 0.2. The maximum and the minimum intensities (max. intensity and min. intensity) for a peak to be weighted relative to the base peak were 1 and 0.1, respectively, and the corresponding influence of the maximal and minimal intensity were 1 and 0.5, respectively.

### Identification of isolated viruses by MALDI-TOF MS

The identification of the 58 viruses isolated from respiratory tract samples was performed by MALDI BioTyper software with the following parameters setting.

#### Preprocessing

Lower Bound 2000 Da, Upper Bound 11,200 Da, resolution 1, Max. peaks 35, threshold 0.015 and S/N 4.

#### Identification

The peaks of main spectra with higher frequency than the set value (frequency threshold for score calculation: 10%) were used for the identification procedure. The mass tolerance for initial peak adjustment before recalibration (max. mass error of the raw spectrum) was 1000 ppm. The desired mass tolerance for any peak after adjusting (des. mass tolerance of the adjusted spectrum) was 250 ppm. The additional mass tolerance for any peak after adjusting (accepted mass tolerance of a peak) was 600 ppm. The preprocessing parameters of the MSP were used for preprocessing the spectra to be identified. To weight the correlation value of the measured peak intensities *versus* the peak intensities of the MSP, this parameter (intensity correction function) was set to 0.25.

The software calculates an arbitrary unit score value between 0 and 3, indicating the similarity between sample and reference spectra and finally displays the top ten matching database records. The results were displayed into the “Detected species” window as specified by the manufacturer with an identification score value ≥ 2.0 for a reliable identification. In this study scores <2.0 were considered as unreliable.

### Statistical analysis using the ClinProTools software

Data analysis began with raw data pretreatment, including baseline subtraction, normalization of a set of spectra, internal peak alignment using prominent peaks, and a peak picking procedure. Subsequently, all sample data derived from virus infected and uninfected cells were imported into ClinProTools software version 2.2 (Bruker Daltonics, Germany) to carry out statistical analysis in order to discover peaks able to differentiate among the different virus subtypes, as previously described^24^.

## Additional Information

**Publisher's note**: Springer Nature remains neutral with regard to jurisdictional claims in published maps and institutional affiliations.

**How to cite this article**: Calderaro, A. *et al*. Identification of different respiratory viruses, after a cell culture step, by matrix assisted laser desorption/ionization time of flight mass spectrometry (MALDI-TOF MS). *Sci. Rep*. **6**, 36082; doi: 10.1038/srep36082 (2016).

## Supplementary Material

Supplementary Table S1

Supplementary Figure S2

Supplementary Figure S3

## Figures and Tables

**Table 1:** Results by Bruker Daltonics MALDI Biotyper with the created in-house database of respiratory viruses isolated from clinical specimens and identified by conventional diagnostic methods.

Analyte number	Conventional identification by routine dignostic methods	MALDI TOF MS identification	Score value (best match)
1	Influenza virus A	Virus influenza A/B	2.554
2		Virus influenza A/B	2.285
3		Virus influenza A/B	2.095
4		Virus influenza A/B	2.000
5		Virus influenza A/B	2.000
6		Virus influenza A/B	2.055
7		Virus influenza A/B	2.250
8		Virus influenza A/B	2.360
1	Influenza virus B	Virus influenza A/B	2.401
2		Virus influenza A/B	2.317
3		Virus influenza A/B	2.200
4		Virus influenza A/B	2.298
5		Virus influenza A/B	2.238
6		Virus influenza A/B	2.000
7		Virus influenza A/B	2.036
8		Virus influenza A/B	2.000
1	Adenovirus	Adenovirus	2.029
2		Adenovirus	2.030
3		Adenovirus	2.492
4		Adenovirus	2.039
5		Adenovirus	2.017
6		Adenovirus	2.058
7		Adenovirus	2.079
8		Adenovirus	2.076
1	Parainfluenza virus type 1	Parainfluenza virus	2.462
1	Parainfluenza virus type 2	Parainfluenza virus	2.167
2		Parainfluenza virus	2.129
1	Parainfluenza virus type 3	Parainfluenza virus	2.603
2		Parainfluenza virus	2.558
3		Parainfluenza virus	2.735
4		Parainfluenza virus	2.754
5		Parainfluenza virus	2.117
6		Parainfluenza virus	2.241
7		Parainfluenza virus	2.404
8		Parainfluenza virus	2.337
1	Respiratory syncytial virus	Respiratory syncytial virus	2.542
2		Respiratory syncytial virus	2.491
3		Respiratory syncytial virus	2.366
4		Respiratory syncytial virus	2.245
5		Respiratory syncytial virus	2.455
6		Respiratory syncytial virus	2.446
7		Respiratory syncytial virus	2.660
8		Respiratory syncytial virus	2.543
9		Respiratory syncytial virus	2.387
1	Echovirus type 30	Echovirus type 30	2.295
2		Echovirus type 30	2.727
3		Echovirus type 30	2.690
4		Echovirus type 30	2.614
5		Echovirus type 30	2.695
6		Echovirus type 30	2.651
7		Echovirus type 30	2.635
8		Echovirus type 30	2.662
1	Cytomegalovirus	Cytomegalovirus	2.256
2		Cytomegalovirus	2.269
3		Cytomegalovirus	2.339
4		Cytomegalovirus	2.427
5		Cytomegalovirus	2.380
1	Metapneumovirus	Metapneumovirus	2.057

**Table 2:** ClinProt Peak Statistic of the type 2 and 5 adenovirus reference strains infected cell cultures used to create MSP.

Mass (m/z)	*p*-value	Ave Adenovirus type 2	Ave Adenovirus type 5	SD Adenovirus type 2	SD Adenovirus type 5
2341	<0.000001	0.4	12.76	0.06	0.91
2355	<0.000001	1.01	13.43	0.07	1.04
2383	0.000047	19.49	2.64	1.71	0.22
2397	0.0000696	13.56	3.68	1.35	0.45
2369	0.000158	15.45	3.37	1.74	0.24

Data derived from the Peak Statistic report *ClinProtStatistic.xml* created using the ClinProTools software. The table shows the five discriminating peaks picked in peak calculation. The mass (*m/z*) value, the *p*-value, the peak area/intensity average (Ave) of type 2 and 5 adenoviruses and the standard deviation of the peak area/intensity average (SD) of type 2 and 5 adenoviruses are displayed for each peak.

**Table 3:** ClinProt Peak Statistic of the adenoviruses isolated from clinical samples and the reference strains infected cell cultures.

Mass (m/z)	*p*-value	Ave ADV 2	Ave ADV 5	Ave 1	Ave 2	Ave 3	Ave 4	Ave 5	Ave 6	Ave 7	Ave 8	SD ADV 2	SD ADV 5	SD1	SD2	SD3	SD4	SD5	SD6	SD7	SD8
2341	<0.000001	0.4	12.76	0.33	0.53	0.42	0.4	0.57	0.42	0.61	0.60	0.06	0.91	0.06	0.09	0.13	0.07	0.13	0.06	0.18	0.16
2356	<0.000001	1.01	13.43	0.45	0.78	1.16	0.62	0.7	0.55	0.62	0.58	0.07	1.04	0.07	0.17	0.15	0.2	0.1	0.09	0.75	0.35
2383	<0.000001	19.49	2.64	16.23	17.89	15.6	19.73	13.36	11.33	12.73	14.65	1.71	0.22	2.3	1.94	1.03	0.95	1.88	2.34	3.75	1.25
2369	<0.000001	15.45	3.37	8.96	10.56	10.18	10.32	9.65	7.37	9.54	9.96	1.74	0.24	1.39	1.1	0.8	0.55	2.94	0.13	0.17	0.65
2397	<0.000001	13.56	3.68	7.05	7.61	8.43	8.62	7.61	7.55	8.58	8.52	1.35	0.45	0.74	0.48	0.29	0.96	1.08	2.1	1.65	2.35

The data derived from the Peak Statistic report *ClinProtStatistic.xml* created using the ClinProTools software. The table shows the three discriminating peaks picked in peak calculation. The mass (*m/z*) value, the *p*-value, the peak area/intensity average of type 2 and 5 adenovirus reference strains (Ave ADV 2 and Ave ADV 5, respectively) and of adenoviruses isolated from eight biological samples (Ave 1, 2, 3, 4, 5, 6, 7 and 8, respectively), and the standard deviation of the peak area/intensity average of the six classes (type 2 and 5 adenovirus reference strains and adenoviruses isolated from eight biological samples: SD ADV 2, SD ADV 5, SD1, SD2, SD3, SD4, SD5, SD6, SD7, and SD8, respectively) are displayed for each peak.

**Table 4:** ClinProt Peak Statistic of the types 1, 2 and 3 of parainfluenza virus reference strains infected cell cultures used to create MSP.

Mass (m/z)	*p*-value	Ave Parainfluenza type 1	Ave Parainfluenza type 2	Ave Parainfluenza type 3	SD Parainfluenza type 1	SD Parainfluenza type 2	SD Parainfluenza type 3
6893	<0.000001	0.98	11.57	21.44	0.11	0.63	1.61
6952	<0.000001	0.62	4	8.72	0.08	0.29	0.16
7006	<0.000001	0.95	5.71	16.92	0.26	0.55	1.63
6275	0.0000107	2.16	8.23	2.05	0.29	0.7	0.58
4230	0.0000181	20.73	1.73	0.2	2.19	0.1	0.05
5655	0.0000456	1.45	5	10.01	0.08	0.86	1.59
6647	0.000144	2.39	11.6	3.28	0.1	1.43	1.25
4085	0.00157	20.54	1.22	0.24	2.83	0.18	0.05

Data derived from the Peak Statistic report *ClinProtStatistic.xml* created using the ClinProTools software. The table shows the eight discriminating peaks picked in peak calculation. The mass (*m/z*) value, the *p*-value, the peak area/intensity average (Ave) of types 1, 2 and 3 of parainfluenza and the standard deviation of the peak area/intensity average (SD) of types 1, 2 and 3 of parainfluenza virus are displayed for each peak.
