# Supplementary material for: Identification of different respiratory viruses, after a cell culture step, by matrix assisted laser desorption/ionization time of flight mass spectrometry (MALDI-TOF MS)
Source: Sci Rep. 2016 Oct 27;6:36082. doi: 10.1038/srep36082 (PMC5081539; doi:10.1038/srep36082)
Supplement: Supplementary Table S1 [file srep36082-s1.pdf]

# IDENTIFICATION OF DIFFERENT RESPIRATORY VIRUSES, AFTER A CELL CULTURE STEP, BY MATRIX ASSISTED LASER DESORPTION/IONIZATION TIME OF FLIGHT MASS SPECTROMETRY (MALDI-TOF MS)

Adriana Calderaro, Maria Cristina Arcangeletti, Isabella Rodighiero, Mirko Buttrini, Sara Montecchini, Rosita Vasile Simone, Maria Cristina Medici, Carlo Chezzi, Flora De Conto

*Department of Clinical and Experimental Medicine – Unit of Microbiology and Virology - University of Parma – Parma, Italy*

**Table S1. Peaks list of all reference virus infected cell culture, with a 100% frequency in all replicates, in the molecular weight range 2000 – 11,200 Da.**

| Virus | Mass (Da) | S/N | Peak intensity (arbitrary unit) | Peak area |
|---|---|---|---|---|
| Human influenza A Neqas 1218 | 3325 | 3.9 | 907 | 5282 |
| | 3948 | 12.4 | 2896 | 25729 |
| | 4002 | 5 | 1176 | 10085 |
| | 4591 | 13.9 | 3155 | 25665 |
| | 4595 | 12.7 | 2888 | 22001 |
| | 4667 | 8.6 | 1961 | 16335 |
| | 4818 | 12.4 | 2893 | 27219 |
| | 5652* | 12.9 | 3037 | 37802 |
| | 5673 | 8.6 | 2016 | 17396 |
| | 6275* | 4.8 | 1063 | 10242 |
| | 6647* | 6.4 | 1396 | 13072 |
| | 6889* | 39.4 | 8467 | 122509 |
| | 6949 | 15.7 | 3351 | 30775 |
| | 7002* | 27.6 | 5945 | 85128 |
| | 7013* | 22.2 | 4782 | 62112 |
| | 7097* | 7.8 | 1623 | 14473 |
| | 7258 | 7.1 | 1466 | 14482 |
| | 10643 | 8.3 | 1236 | 13744 |
| Human influenza A ATCC VR219 | 4592 | 14.1 | 2519 | 17981 |
| | 4596 | 11.5 | 2037 | 14957 |
| | 4669 | 6.6 | 1149 | 8491 |
| | 4667 | 5.5 | 961 | 4907 |
| | 5654* | 16.6 | 2964 | 35803 |
| | 5674 | 10.2 | 1822 | 15078 |
| | 6889* | 59.3 | 11721 | 226095 |
| | 6895 | 50.2 | 9949 | 141152 |
| | 6952 | 21.3 | 4174 | 37539 |
| | 7005* | 27.9 | 5394 | 70769 |
| | 7016* | 22 | 4260 | 56054 |
| | 7101 | 6.1 | 1228 | 7518 |
| | 7261 | 4.2 | 763 | 5632 |
| | 10650 | 6.6 | 1303 | 14530 |
| | 10890* | 8 | 1576 | 16757 |
| Human influenza B Neqas 7260 | 2078 | 5.6 | 1922 | 10023 |
| | 3137 | 5.1 | 1764 | 9414 |
| | 3323 | 4.3 | 1535 | 7816 |
| | 3456 | 9.1 | 3291 | 22634 |
| | 3948 | 8.6 | 3329 | 33066 |

| | | | | |
|---|---|---|---|---|
| | 4592 | 10.4 | 3671 | 32154 |
| | 4596 | 9.4 | 3359 | 33082 |
| | 4669 | 6 | 2173 | 20424 |
| | 4676 | 7.1 | 2493 | 19741 |
| | 6276* | 10.7 | 4265 | 47590 |
| | 6648* | 9.5 | 3823 | 41556 |
| | 6888* | 36 | 13148 | 273979 |
| | 6894 | 31.7 | 11734 | 220372 |
| | 7004* | 20.4 | 7602 | 112146 |
| | 7015* | 16.4 | 6190 | 95808 |
| | 7099 | 5.2 | 1955 | 24281 |
| | 7259 | 4.4 | 1635 | 17588 |
| | 10646 | 6.2 | 1679 | 24962 |
| | 10887* | 10.1 | 2726 | 61316 |
| Parainfluenza virus type 1 ATCC VR-907 | 2413 | 5 | 8387 | 34403 |
| | 2505 | 5.4 | 9046 | 42116 |
| | 2688 | 4.6 | 7528 | 46826 |
| | 3101 | 2.8 | 4270 | 20931 |
| | 3182 | 4.3 | 6532 | 28192 |
| | 4083 | 9.7 | 12486 | 82799 |
| | 4228 | 10 | 12590 | 86704 |
| | 4243 | 2.4 | 3058 | 7997 |
| | 4357 | 3.4 | 3991 | 20556 |
| | 4432 | 2.9 | 3512 | 21702 |
| | 4477 | 3.4 | 3891 | 25924 |
| | 4774 | 5.3 | 5721 | 37244 |
| | 4984** | 2.7 | 2640 | 16357 |
| | 6645** | 2.9 | 1620 | 13091 |
| | 8446 | 6.2 | 1821 | 22547 |
| Parainfluenza virus type 2 ATCC VR-92 | 3136** | 6.2 | 6866 | 16543 |
| | 3271 | 3.6 | 5146 | 11178 |
| | 3455** | 16 | 13419 | 57991 |
| | 4588 | 3.6 | 4684 | 10135 |
| | 4595 | 5.9 | 6259 | 18880 |
| | 4936** | 4 | 4941 | 16202 |
| | 4963** | 7.1 | 6972 | 28319 |
| | 5471 | 3.1 | 4014 | 14025 |
| | 5652 | 5.9 | 5736 | 21978 |
| | 5673 | 2.8 | 3822 | 8818 |
| | 6274** | 10.5 | 8115 | 48236 |
| | 6543 | 5.8 | 5217 | 28063 |
| | 6893 | 16.6 | 10741 | 59794 |
| | 7272 | 5.8 | 4568 | 16236 |
| | 7411 | 3.1 | 3130 | 7286 |
| | 7509 | 8.5 | 5642 | 24738 |

| | | | | |
|---|---|---|---|---|
| | 8562 | 9 | 4716 | 35657 |
| Parainfluenza virus type 3 Neqas 1216 | 3138** | 8.6 | 3274 | 18837 |
| | 3272 | 2.9 | 1108 | 6217 |
| | 3324** | 8.5 | 3296 | 19498 |
| | 3460** | 15.1 | 5798 | 40760 |
| | 3505 | 3.8 | 1451 | 9234 |
| | 3820 | 2.3 | 930 | 6366 |
| | 5477 | 4.7 | 2107 | 24317 |
| | 6278** | 16.8 | 7534 | 77197 |
| | 6546 | 5.2 | 2278 | 26222 |
| | 6650** | 16.7 | 7115 | 72873 |
| | 7005** | 8.4 | 3720 | 43522 |
| | 7514 | 3 | 1233 | 10942 |
| | 10905 | 6.2 | 1711 | 26695 |
| Respiratory syncytial virus Neqas 1217 | 3457*** | 19.4 | 2581 | 18558 |
| | 3770 | 7.4 | 1067 | 6456 |
| | 4594 | 17.1 | 2708 | 25576 |
| | 4670 | 14.1 | 2247 | 25957 |
| | 4939 | 7.9 | 1247 | 11138 |
| | 4966*** | 7.8 | 1237 | 10831 |
| | 6278*** | 5.7 | 963 | 9333 |
| | 6896*** | 61.5 | 10906 | 213725 |
| | 7006*** | 50.5 | 8951 | 198430 |
| | 7096 | 12.9 | 2229 | 31301 |
| | 7263 | 9.8 | 1690 | 21396 |
| | 10641 | 15.5 | 2097 | 48234 |
| | 10891 | 19.3 | 2616 | 51717 |
| Adenovirus type 5 ATCC VR-1516 | 2188*** | 6.1 | 3351 | 7914 |
| | 2342 | 21.1 | 7844 | 30919 |
| | 2356 | 22.1 | 8161 | 32592 |
| | 2438 | 86.6 | 24660 | 144329 |
| | 2460 | 21.1 | 7718 | 34899 |
| | 2546 | 14 | 5690 | 18049 |
| | 2567 | 5 | 3195 | 8125 |
| | 2601 | 5.1 | 3199 | 8246 |
| | 2617 | 7.1 | 3754 | 9456 |
| | 3035 | 60.9 | 17399 | 105590 |
| | 3154 | 11.3 | 4464 | 22030 |
| | 3624 | 44.6 | 11843 | 71787 |
| Adenovirus type 2 Neqas 9054 | 2369 | 23.38 | 25413 | 112146 |
| | 2383 | 29.46 | 31098 | 132759 |
| | 2397 | 20.52 | 22708 | 100016 |
| | 2411 | 15.96 | 18423 | 79181 |
| | 2438 | 59.68 | 58164 | 380413 |
| | 2466 | 28.4 | 30001 | 141544 |

| | | | | |
|---|---|---|---|---|
| | 2545 | 4.52 | 7688 | 24119 |
| | 2573 | 3.82 | 7009 | 18122 |
| | 3035 | 59.82 | 57806 | 400787 |
| | 3050 | 7,3 | 9946 | 48591 |
| | 3151 | 5,7 | 8437 | 41200 |
| | 3280 | 3,4 | 6298 | 20300 |
| | 3323 | 5,3 | 8077 | 24795 |
| | 3337 | 5,5 | 8197 | 29172 |
| | 5653 | 10,5 | 13009 | 90298 |
| | 5667 | 11 | 13562 | 85521 |
| | 6275*** | 11 | 13639 | 106725 |
| | 6303 | 8 | 10771 | 79641 |
| | 6648*** | 13 | 16438 | 117103 |
| | 6675 | 10,7 | 14347 | 108891 |
| | 7004*** | 21,1 | 22946 | 185871 |
| | 7017 | 25,4 | 26727 | 287679 |
| | 7030 | 21,5 | 23390 | 338884 |
| | 7315 | 3,6 | 7781 | 21868 |
| | 10971 | 13 | 9954 | 56227 |
| Cytomegalovirus ATCC VR-977 | 2747 | 2.4 | 2475 | 10104 |
| | 2947 | 3.3 | 3639 | 18995 |
| | 3326 | 3.2 | 3568 | 19759 |
| | 3421 | 4.2 | 4592 | 24199 |
| | 3455$ | 10.6 | 11619 | 81385 |
| | 3493 | 4.8 | 5287 | 36314 |
| | 3506 | 3.7 | 4120 | 22870 |
| | 4075$ | 10.4 | 11374 | 84141 |
| | 4098 | 2.6 | 2844 | 11921 |
| | 4139$ | 9.3 | 10227 | 74504 |
| | 4207$ | 4.2 | 4616 | 26161 |
| | 4411$ | 6.3 | 6917 | 48986 |
| | 4936$ | 3.3 | 3565 | 23506 |
| | 4962$ | 3 | 3235 | 23024 |
| | 5304 | 3.7 | 3904 | 28414 |
| | 5491 | 4.3 | 4487 | 35343 |
| | 5896$ | 11.9 | 11739 | 134276 |
| | 6543$ | 4.4 | 3952 | 35529 |
| | 6652 | 4.5 | 3894 | 38426 |
| | 6843 | 7.3 | 6290 | 63876 |
| | 6929 | 4.7 | 3942 | 35701 |
| | 7008$ | 7.2 | 5785 | 54930 |
| | 7993 | 5.1 | 3440 | 29593 |
| | 8426 | 5.3 | 3600 | 44873 |
| | 2188 | 8.5 | 12953 | 58414 |

| | | | | |
|---|---|---|---|---|
| Echovirus type 30 ATCC VR-322 | 2203 | 3.4 | 5597 | 27571 |
| | 2812 | 3 | 5021 | 23476 |
| | 3267 | 2.5 | 4149 | 17633 |
| | 3421 | 2.4 | 3876 | 17488 |
| | 3493 | 3 | 4846 | 26153 |
| | 3505 | 2.9 | 4710 | 29837 |
| | 4075[$] | 14.7 | 20104 | 176259 |
| | 4138[$] | 11.1 | 16195 | 163306 |
| | 4207[$] | 7.8 | 11147 | 80119 |
| | 4371 | 4.3 | 6680 | 54299 |
| | 4411[$] | 8.2 | 12215 | 97768 |
| | 4899 | 4.9 | 7154 | 67214 |
| | 4935[$] | 3.4 | 5052 | 38880 |
| | 4963[$] | 4.3 | 6248 | 48299 |
| | 5492 | 7.6 | 10622 | 105569 |
| | 5748 | 3.4 | 4491 | 48414 |
| | 5802 | 3.2 | 4286 | 32725 |
| | 5897[$] | 12.1 | 15416 | 160660 |
| | 5972 | 3 | 3648 | 34510 |
| | 6543[$] | 6.4 | 7058 | 62836 |
| | 6654 | 7 | 7473 | 73265 |
| | 6875 | 5.4 | 5991 | 57639 |
| | 6987 | 6.4 | 6566 | 73305 |
| | 7011[$] | 8.5 | 8643 | 114220 |
| | 7809 | 4.4 | 3617 | 44935 |
| | 7995 | 4.7 | 3997 | 45509 |
| | 8426 | 5.2 | 4034 | 45497 |
| | 8546 | 3.6 | 2434 | 22173 |
| | 8563 | 2.9 | 2134 | 22681 |
| | 8791 | 3.6 | 1975 | 24161 |
| | 10218 | 3.7 | 1823 | 20401 |
| Metapneumovirus | 3370 | 46.1 | 10996 | 77484 |
| | 3441 | 52.1 | 12034 | 96025 |
| | 3485** | 59.7 | 13357 | 121780 |
| | 3708 | 12.5 | 3656 | 17107 |

S/N: signal to noise ratio
* peaks present also in MDCK SIAT1 uninfected cells spectrum.
** peaks present also in LLC-MK2 uninfected cells spectrum.
*** peaks present also in Intestine 407 uninfected cells spectrum.
[$] peaks present also in MRC5 uninfected cells spectrum.
