# Supplementary material for: Identification of different respiratory viruses, after a cell culture step, by matrix assisted laser desorption/ionization time of flight mass spectrometry (MALDI-TOF MS)
Source: Sci Rep. 2016 Oct 27;6:36082. doi: 10.1038/srep36082 (PMC5081539; doi:10.1038/srep36082)
Supplement: Supplementary Figure S2 [file srep36082-s2.pdf]

IDENTIFICATION OF DIFFERENT RESPIRATORY VIRUSES, AFTER A CELL CULTURE STEP, BY MATRIX ASSISTED LASER DESORPTION/IONIZATION TIME OF FLIGHT MASS SPECTROMETRY (MALDI-TOF MS)

Adriana Calderaro, Maria Cristina Arcangeletti, Isabella Rodighiero, Mirko Buttrini, Sara Montecchini, Rosita Vasile Simone, Maria Cristina Medici, Carlo Chezzi, Flora De Conto

Department of Clinical and Experimental Medicine – Unit of Microbiology and Virology - University of Parma – Parma, Italy

Figure S2. Statistical analysis performed on spectra sets of different reference strains infected cell cultures obtained in different conditions (different operators, different days).

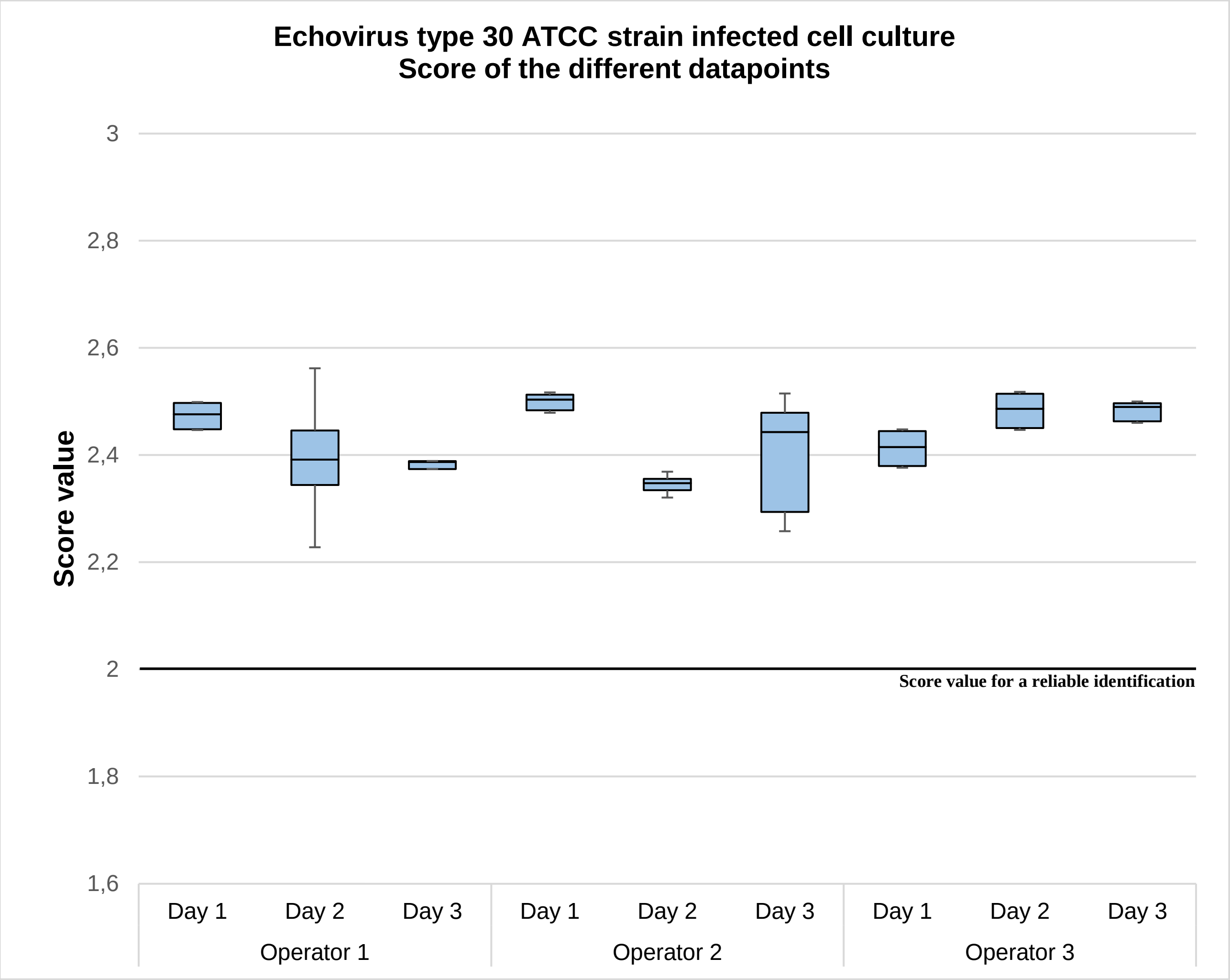

Box plot showing the distribution of score values obtained for the replicates of Echovirus type 30 ATCC strain infected cell culture by three independent operators in three different days.

Box plot explanation: upper horizontal line of box, 75th percentile; lower horizontal line of box, 25th percentile; horizontal bar within box, median; upper horizontal bar outside box, 90th percentile; lower horizontal bar outside box, 10th percentile.

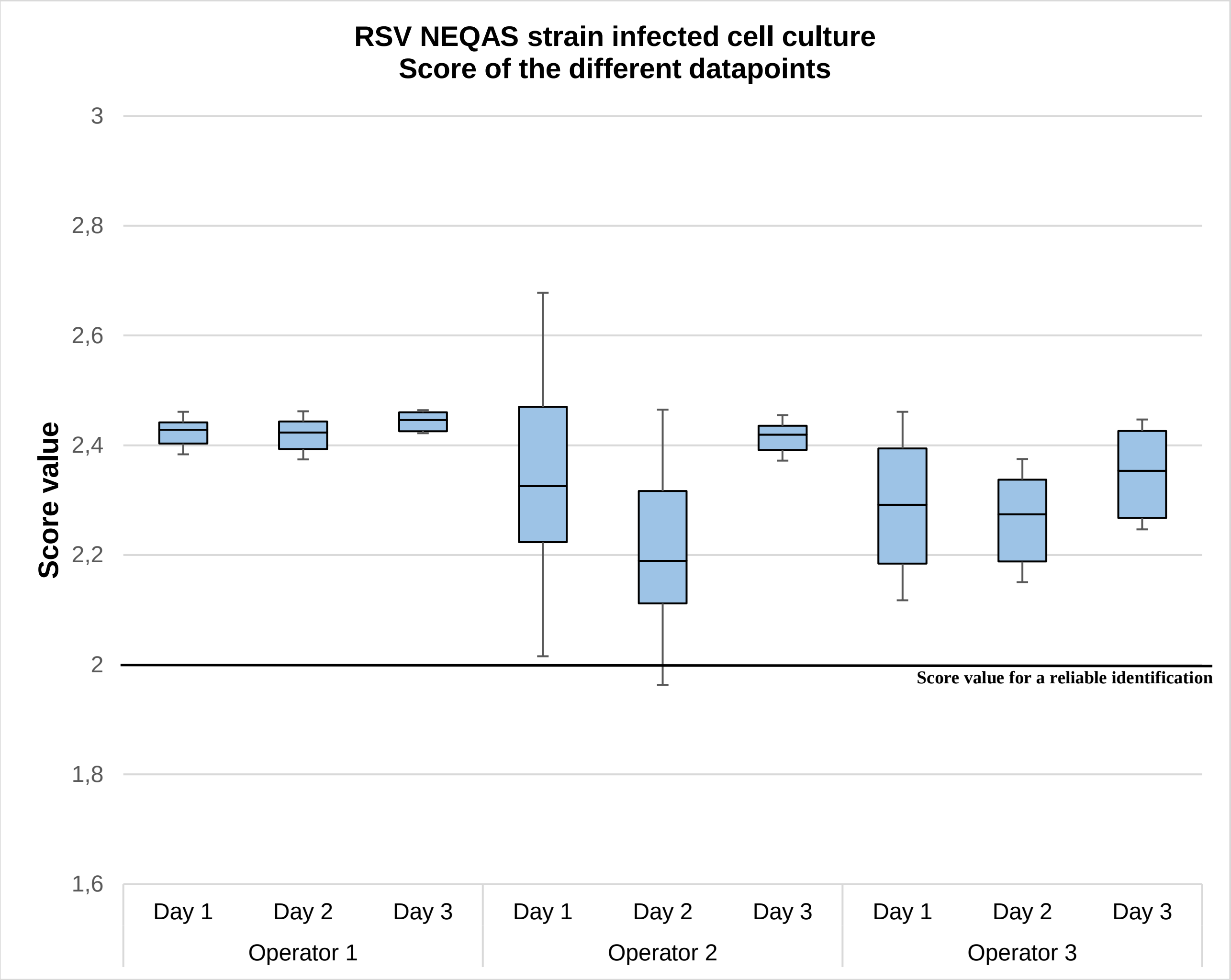

Box plot showing the distribution of score values obtained for the replicates of respiratory syncytial virus (RSV) NEQAS strain infected cell culture by three independent operators in three different days.

Box plot explanation: upper horizontal line of box, 75th percentile; lower horizontal line of box, 25th percentile; horizontal bar within box, median; upper horizontal bar outside box, 90th percentile; lower horizontal bar outside box, 10th percentile.

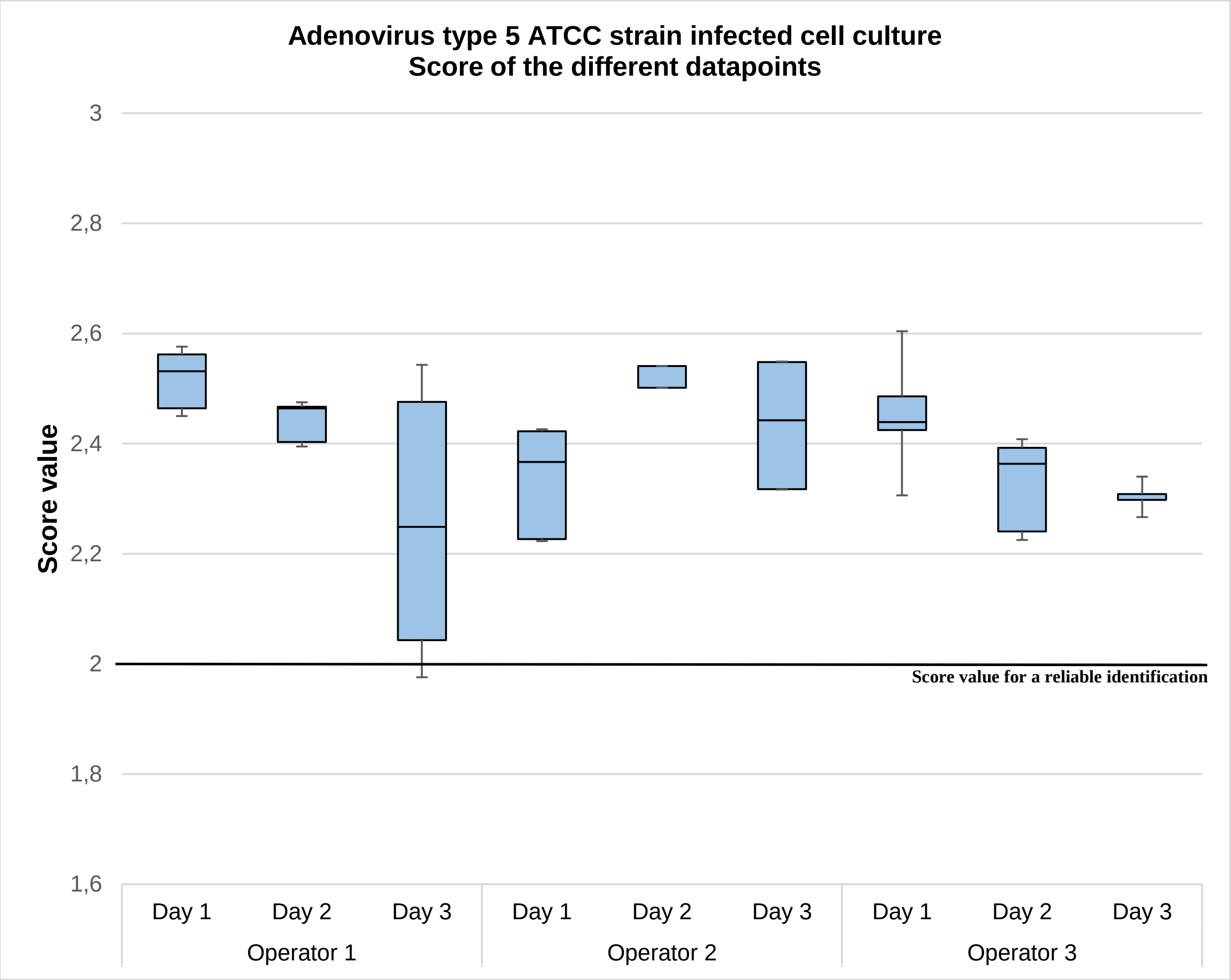

Box plot showing the distribution of score values obtained for the replicates of Adenovirus type 5 ATCC strain infected cell culture by three independent operators in three different days.

Box plot explanation: upper horizontal line of box, 75th percentile; lower horizontal line of box, 25th percentile; horizontal bar within box, median; upper horizontal bar outside box, 90th percentile; lower horizontal bar outside box, 10th percentile.

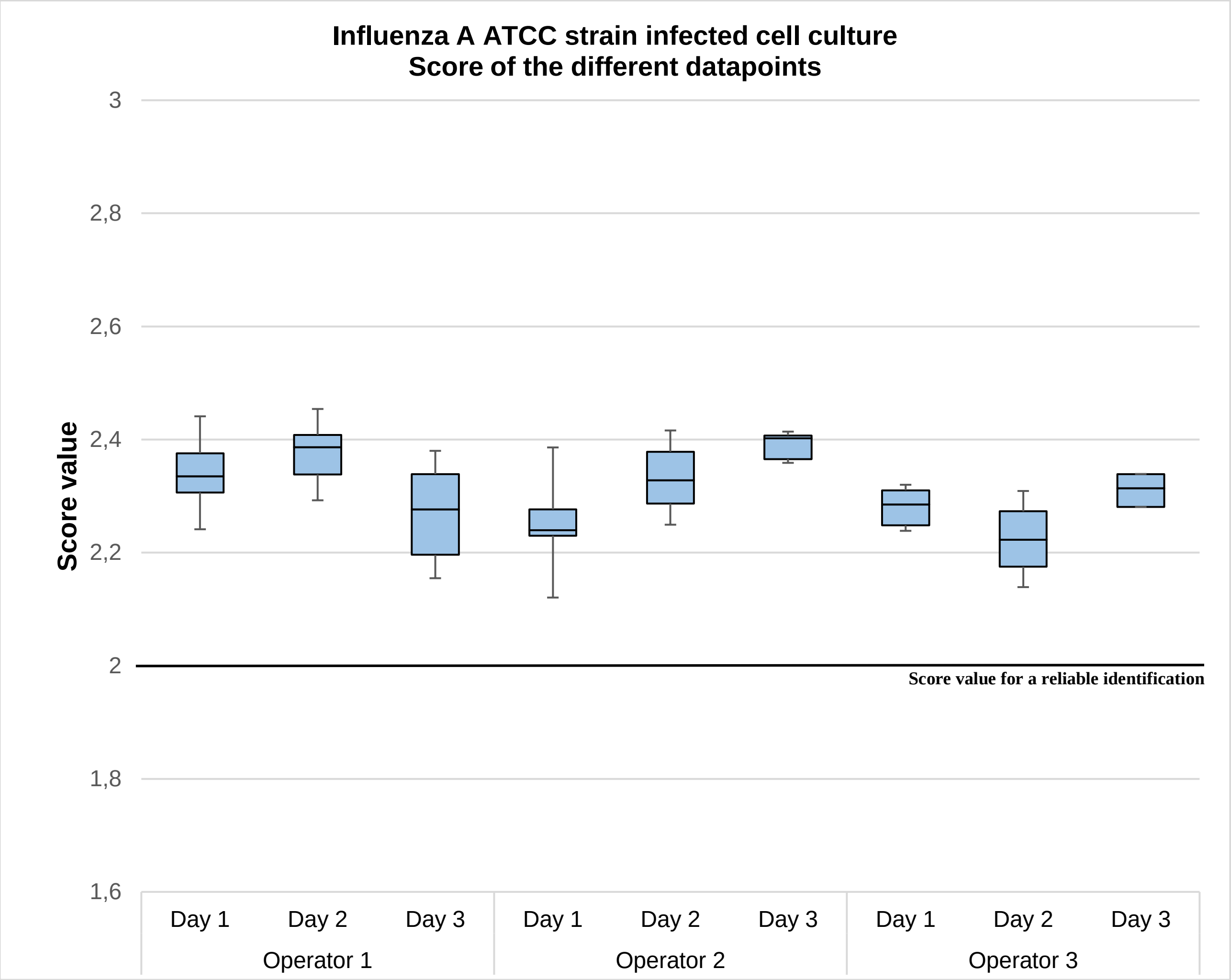

Box plot showing the distribution of score values obtained for the replicates of Influenza A ATCC strain infected cell culture by three independent operators in three different days.

Box plot explanation: upper horizontal line of box, 75th percentile; lower horizontal line of box, 25th percentile; horizontal bar within box, median; upper horizontal bar outside box, 90th percentile; lower horizontal bar outside box, 10th percentile.

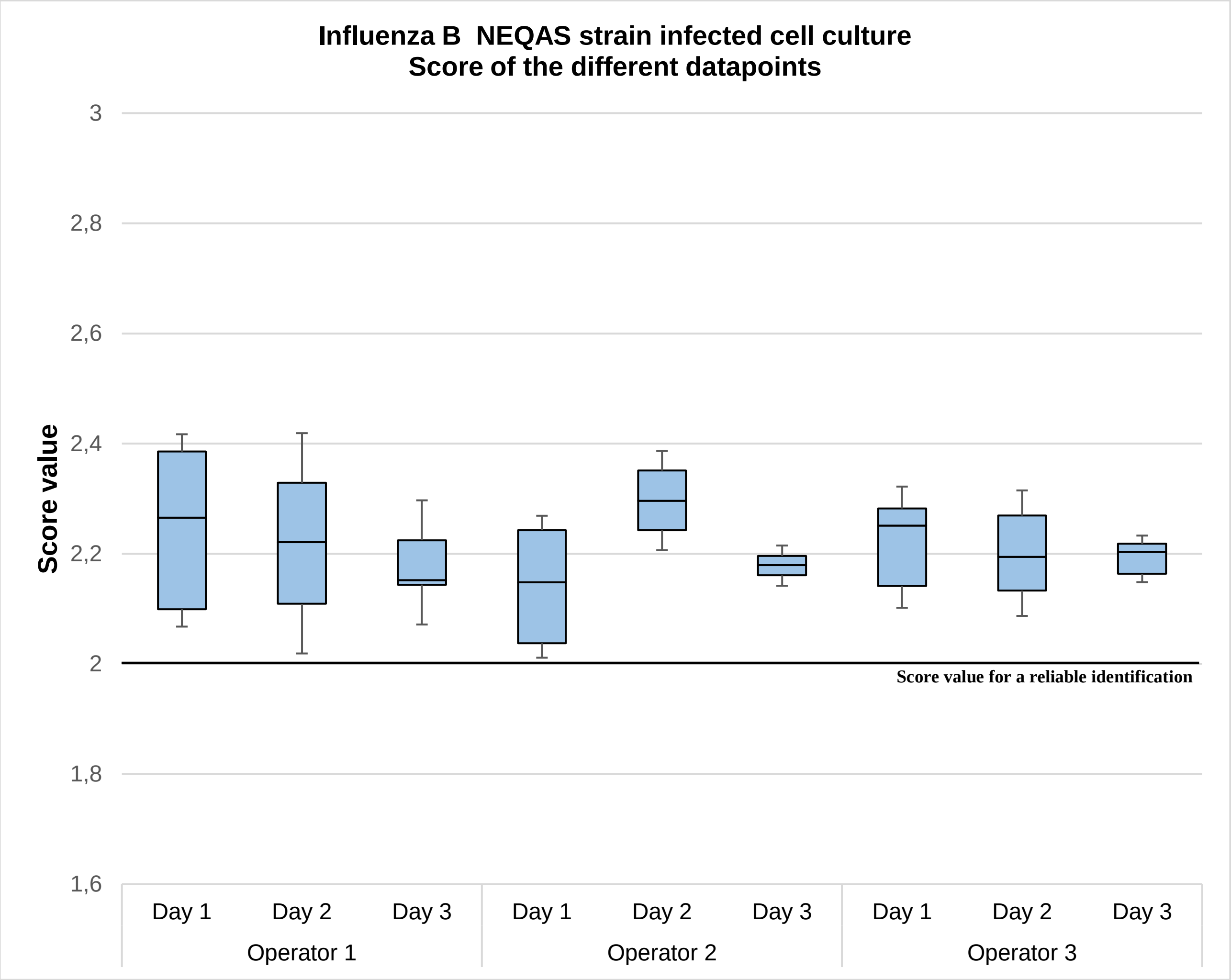

Box plot showing the distribution of score values obtained for the replicates of Influenza B NEQAS strain infected cell culture by three independent operators in three different days.

Box plot explanation: upper horizontal line of box, 75th percentile; lower horizontal line of box, 25th percentile; horizontal bar within box, median; upper horizontal bar outside box, 90th percentile; lower horizontal bar outside box, 10th percentile.

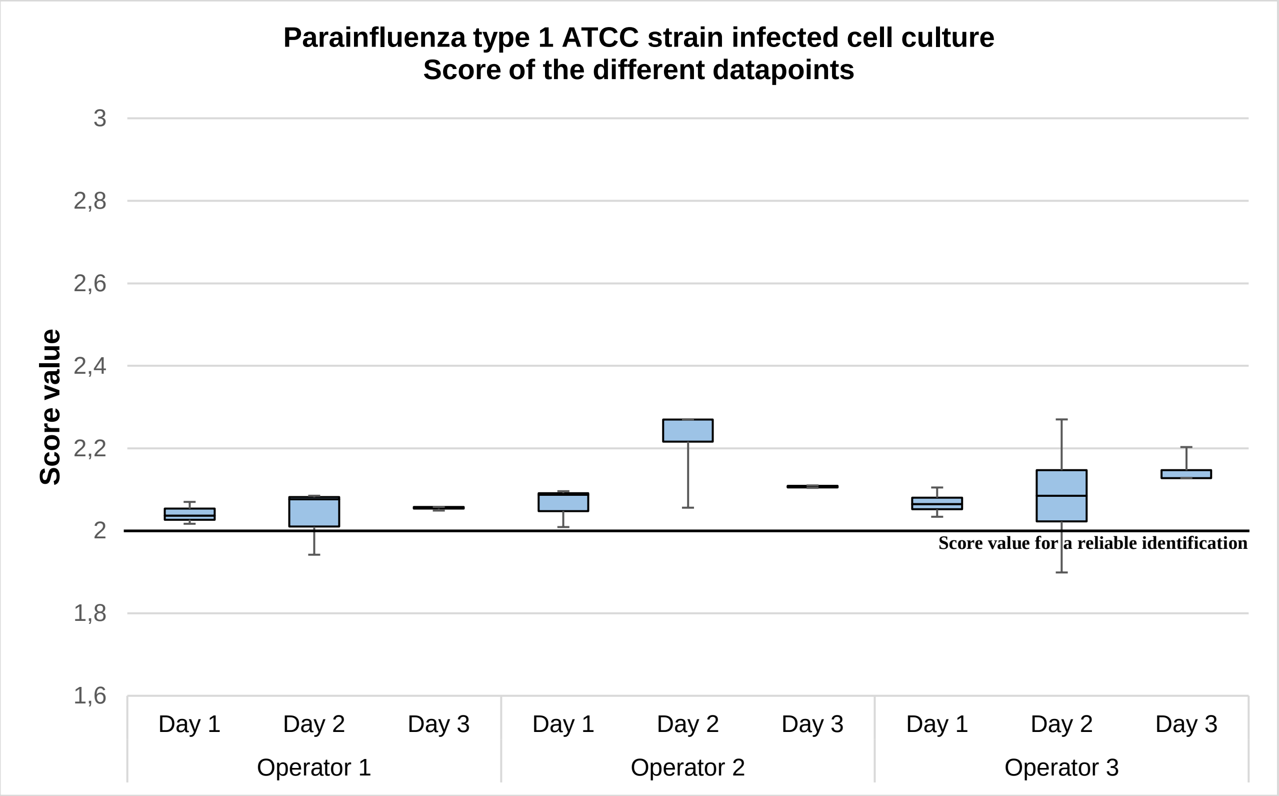

Box plot showing the distribution of score values obtained for the replicates of Parainfluenza type 1 ATCC strain infected cell culture by three independent operators in three different days.

Box plot explanation: upper horizontal line of box, 75th percentile; lower horizontal line of box, 25th percentile; horizontal bar within box, median; upper horizontal bar outside box, 90th percentile; lower horizontal bar outside box, 10th percentile.

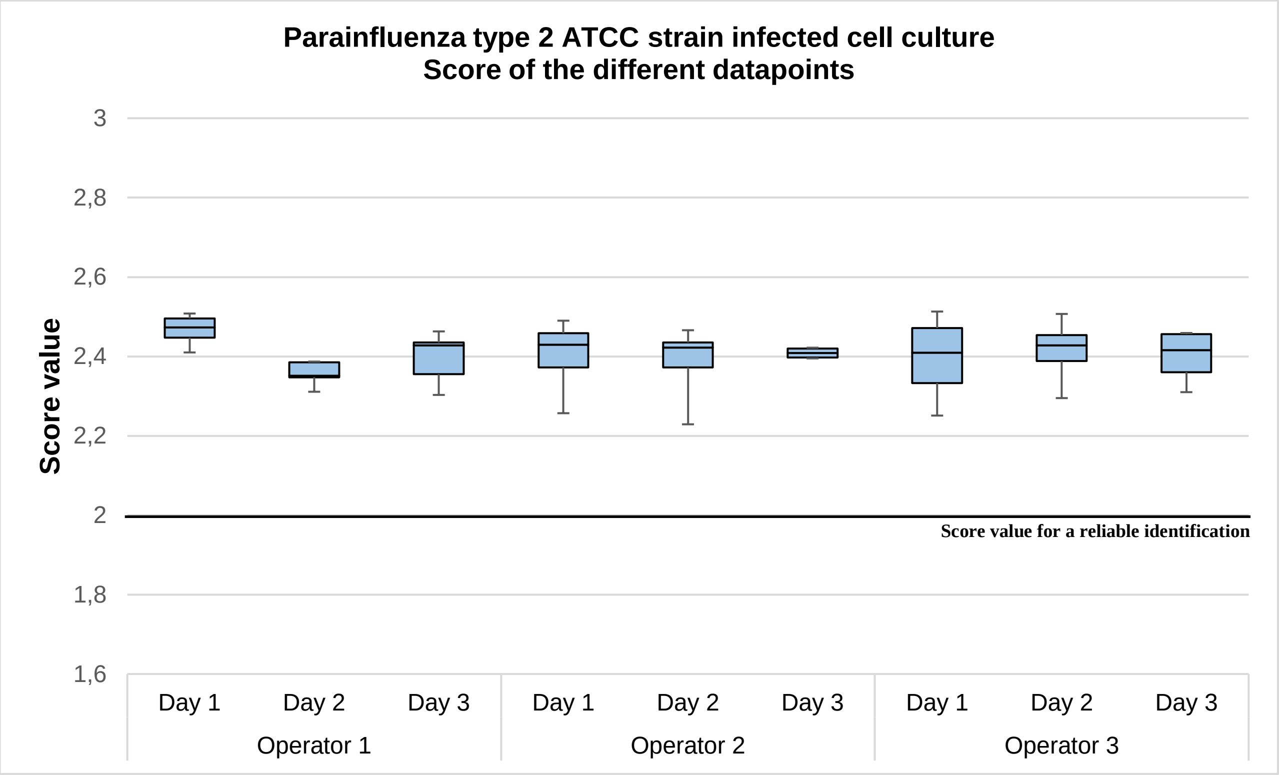

Box plot showing the distribution of score values obtained for the replicates of Parainfluenza type 2 ATCC strain infected cell culture by three independent operators in three different days.

Box plot explanation: upper horizontal line of box, 75th percentile; lower horizontal line of box, 25th percentile; horizontal bar within box, median; upper horizontal bar outside box, 90th percentile; lower horizontal bar outside box, 10th percentile.

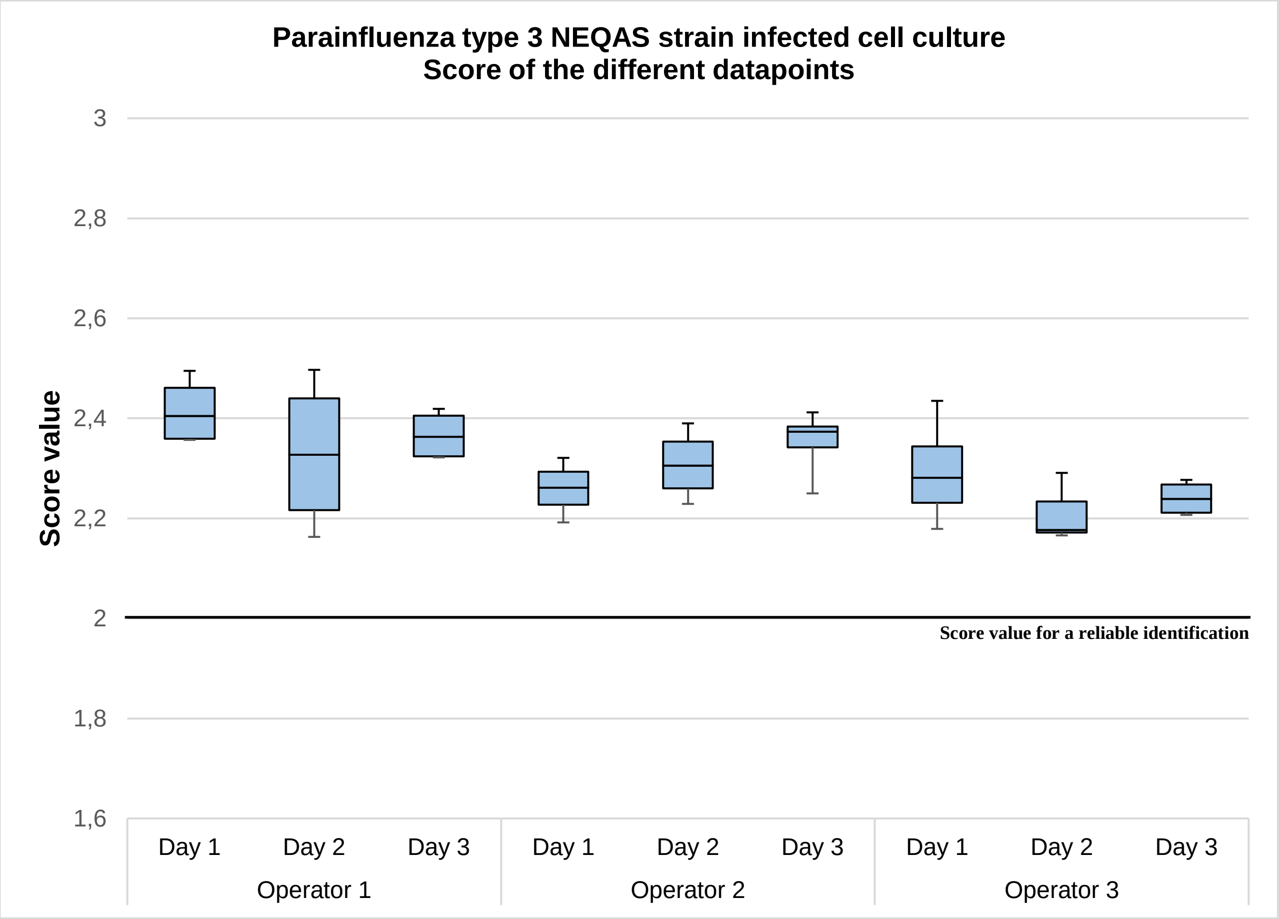

Box plot showing the distribution of score values obtained for the replicates of Parainfluenza type 3 NEQAS strain infected cell culture by three independent operators in three different days.

Box plot explanation: upper horizontal line of box, 75th percentile; lower horizontal line of box, 25th percentile; horizontal bar within box, median; upper horizontal bar outside box, 90th percentile; lower horizontal bar outside box, 10th percentile.

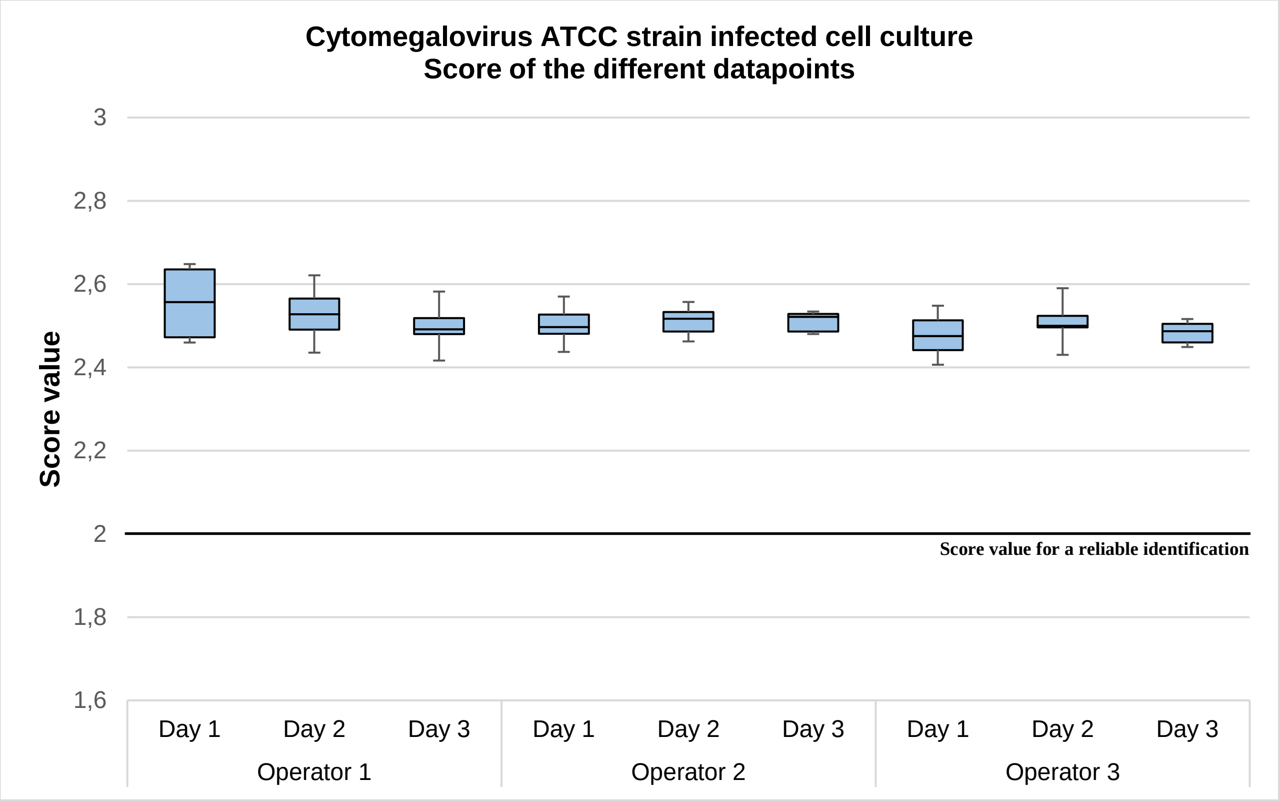

Box plot showing the distribution of score values obtained for the replicates of Cytomegalovirus ATCC strain infected cell culture by three independent operators in three different days.

Box plot explanation: upper horizontal line of box, 75th percentile; lower horizontal line of box, 25th percentile; horizontal bar within box, median; upper horizontal bar outside box, 90th percentile; lower horizontal bar outside box, 10th percentile.

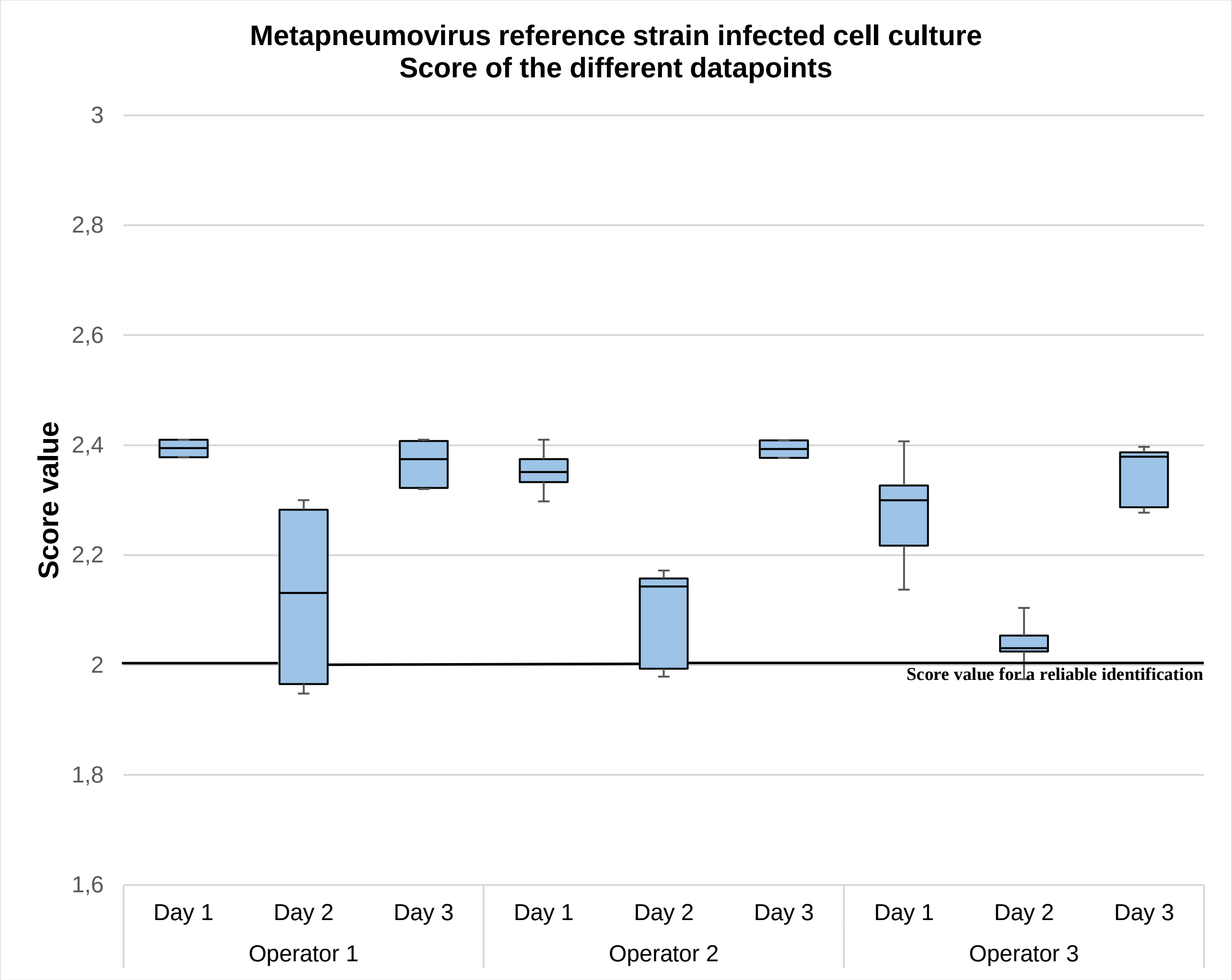

Box plot showing the distribution of score values obtained for the replicates of Metapneumovirus reference strain infected cell culture by three independent operators in three different days.

Box plot explanation: upper horizontal line of box, 75th percentile; lower horizontal line of box, 25th percentile; horizontal bar within box, median; upper horizontal bar outside box, 90th percentile; lower horizontal bar outside box, 10th percentile.
