# Supplementary material for: Identification of different respiratory viruses, after a cell culture step, by matrix assisted laser desorption/ionization time of flight mass spectrometry (MALDI-TOF MS)
Source: Sci Rep. 2016 Oct 27;6:36082. doi: 10.1038/srep36082 (PMC5081539; doi:10.1038/srep36082)
Supplement: Supplementary Figure S3 [file srep36082-s3.pdf]

# IDENTIFICATION OF DIFFERENT RESPIRATORY VIRUSES, AFTER A CELL CULTURE STEP, BY MATRIX ASSISTED LASER DESORPTION/IONIZATION TIME OF FLIGHT MASS SPECTROMETRY (MALDI-TOF MS)

Adriana Calderaro, Maria Cristina Arcangeletti, Isabella Rodighiero, Mirko Buttrini, Sara Montecchini, Rosita Vasile Simone, Maria Cristina Medici, Carlo Chezzi, Flora De Conto

*Department of Clinical and Experimental Medicine – Unit of Microbiology and Virology - University of Parma – Parma, Italy*

**Figure S3. Statistical analysis performed on spectra sets of different clinical strains infected cell cultures obtained in different conditions (different operators, different days).**

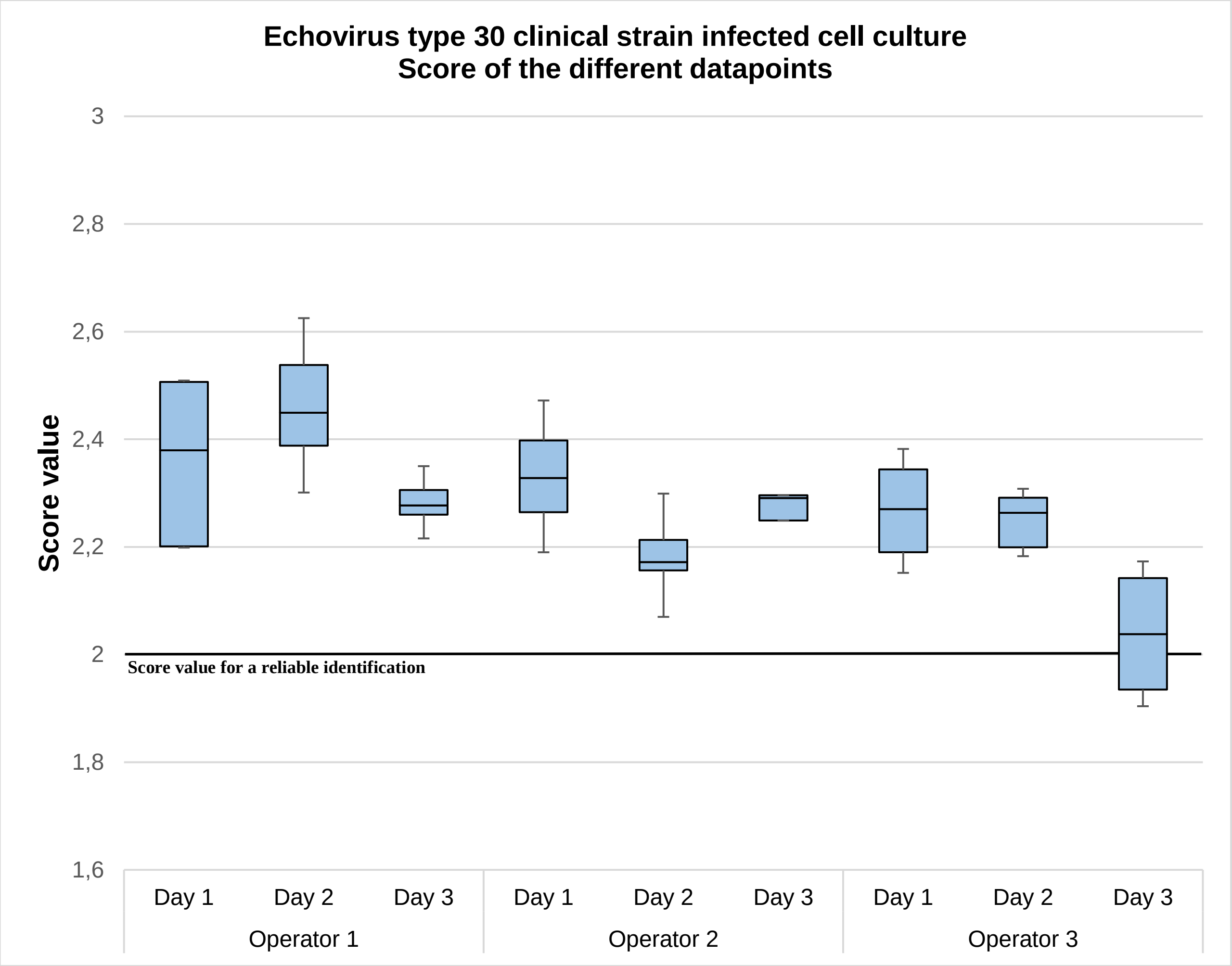


**Box plot showing the distribution of score values obtained for the replicates of Echovirus type 30 clinical strain infected cell culture by three independent operators in three different days.**

Box plot explanation: upper horizontal line of box, 75th percentile; lower horizontal line of box, 25th percentile; horizontal bar within box, median; upper horizontal bar outside box, 90th percentile; lower horizontal bar outside box, 10th percentile.

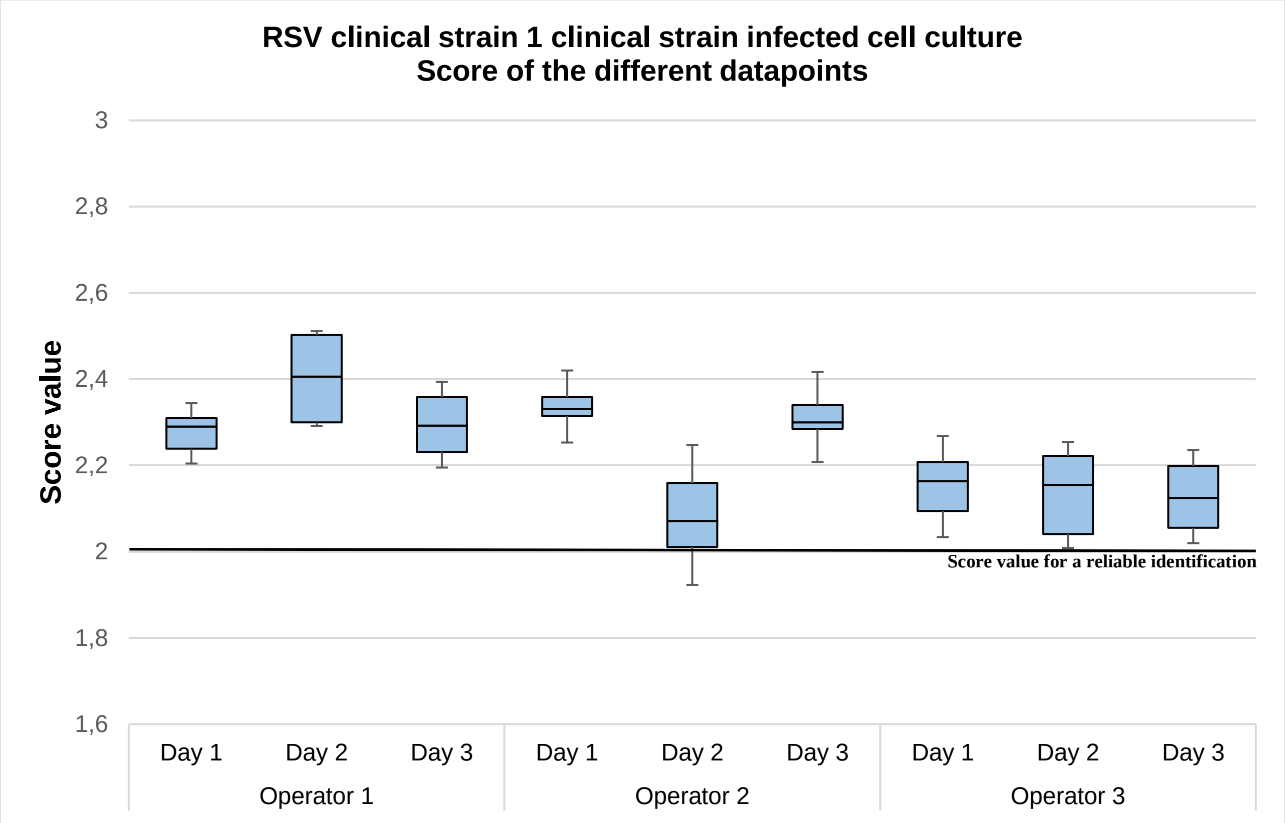


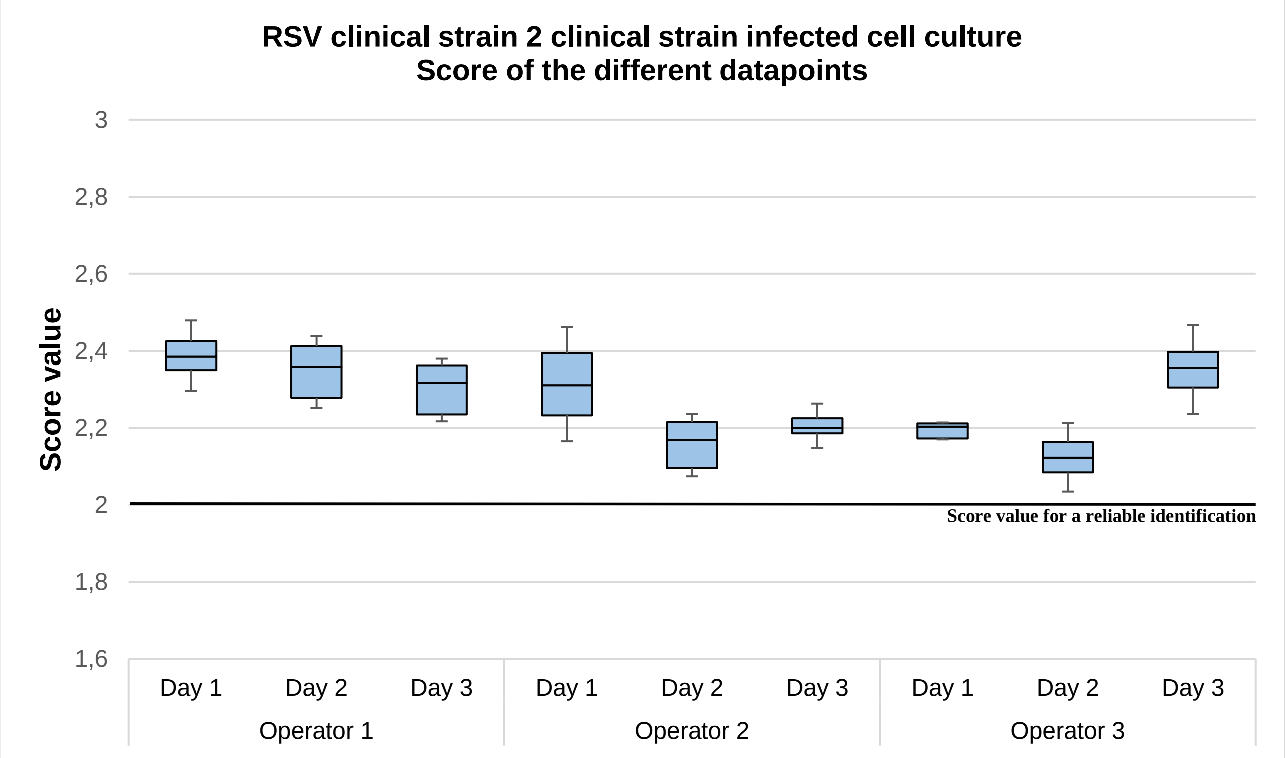


**Box plot showing the distribution of score values obtained for the replicates of 2 respiratory syncytial virus (RSV) clinical strains infected cell cultures by three independent operators in three different days.**

Box plot explanation: upper horizontal line of box, 75th percentile; lower horizontal line of box, 25th percentile; horizontal bar within box, median; upper horizontal bar outside box, 90th percentile; lower horizontal bar outside box, 10th percentile.

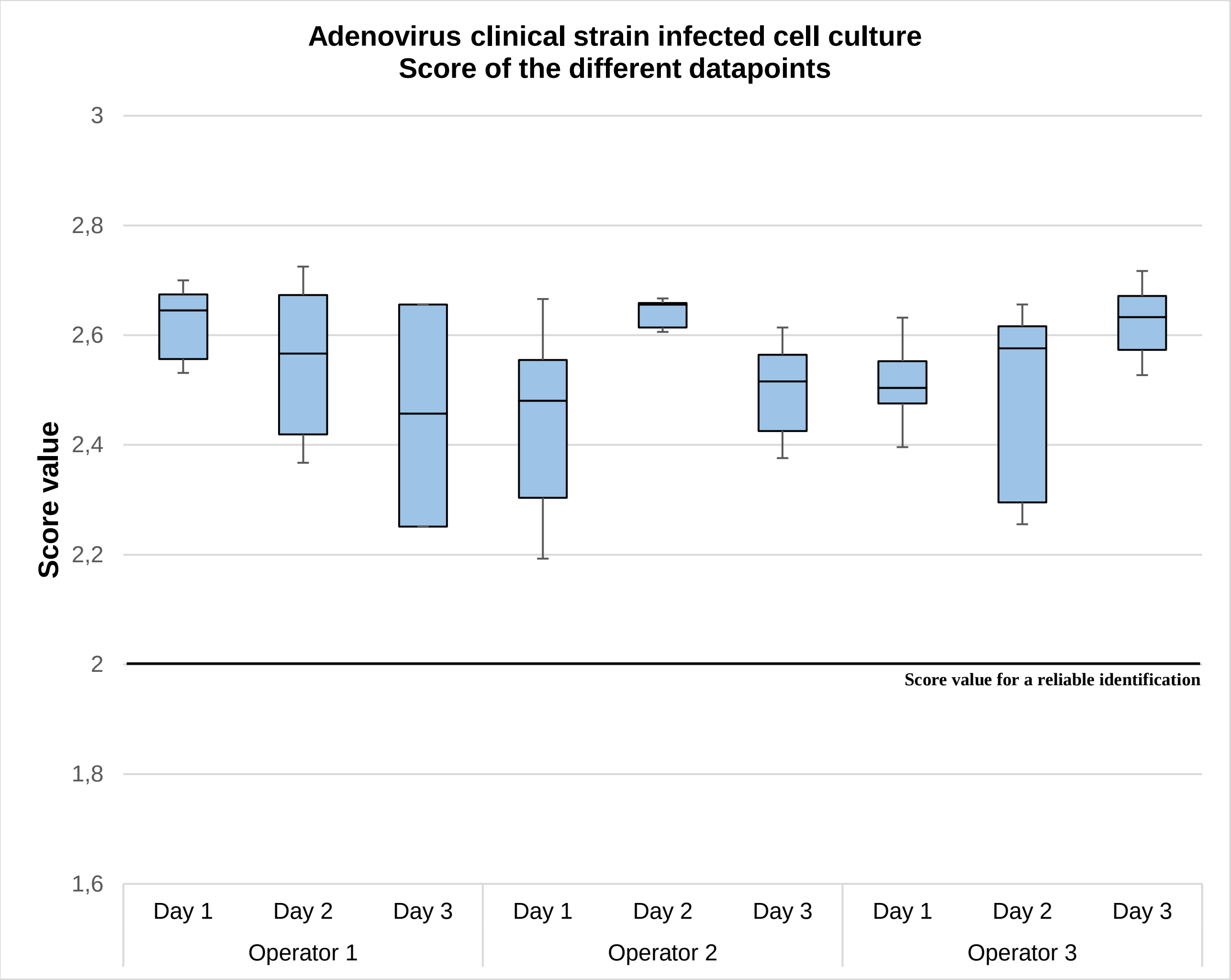


**Box plot showing the distribution of score values obtained for the replicates of Adenovirus clinical strain infected cell culture by three independent operators in three different days.**

Box plot explanation: upper horizontal line of box, 75th percentile; lower horizontal line of box, 25th percentile; horizontal bar within box, median; upper horizontal bar outside box, 90th percentile; lower horizontal bar outside box, 10th percentile.

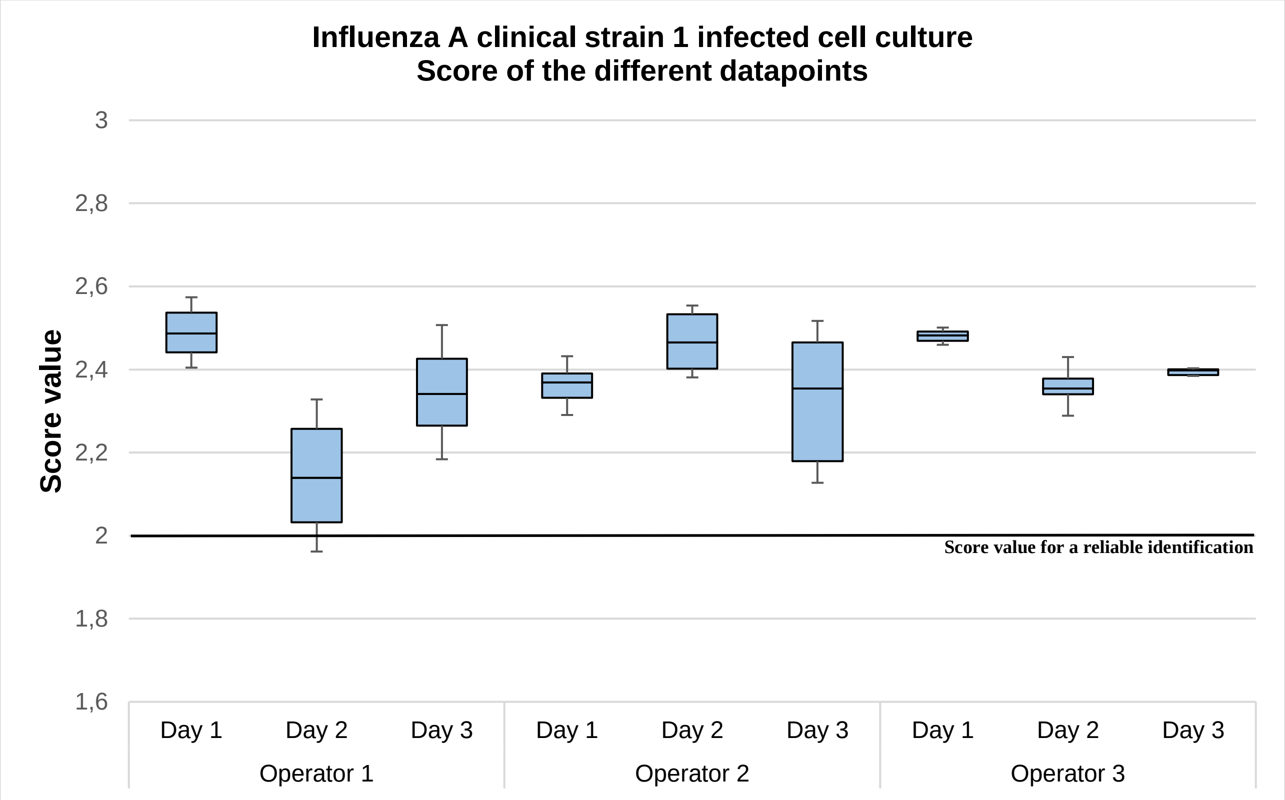


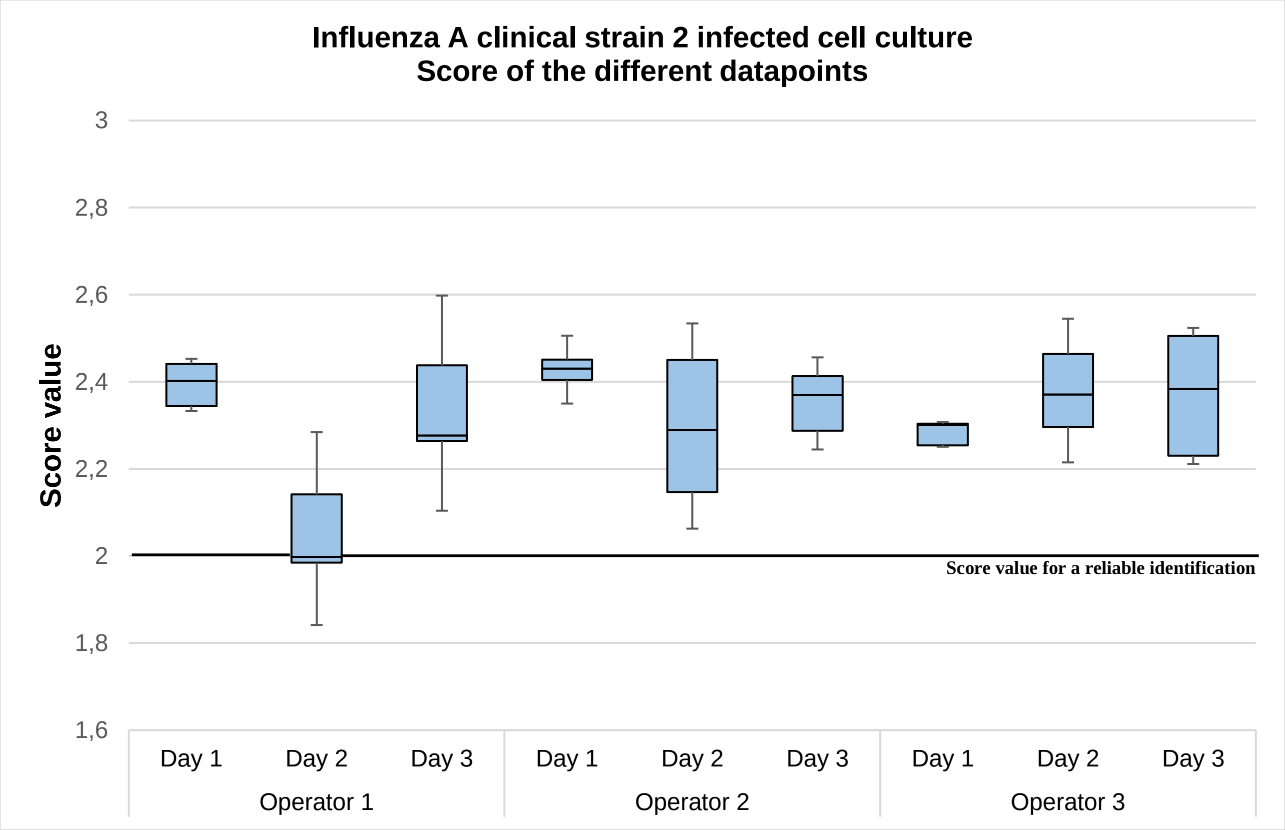


**Box plot showing the distribution of score values obtained for the replicates of 2 Influenza A clinical strains infected cell cultures by three independent operators in three different days.**

Box plot explanation: upper horizontal line of box, 75th percentile; lower horizontal line of box, 25th percentile; horizontal bar within box, median; upper horizontal bar outside box, 90th percentile; lower horizontal bar outside box, 10th percentile.

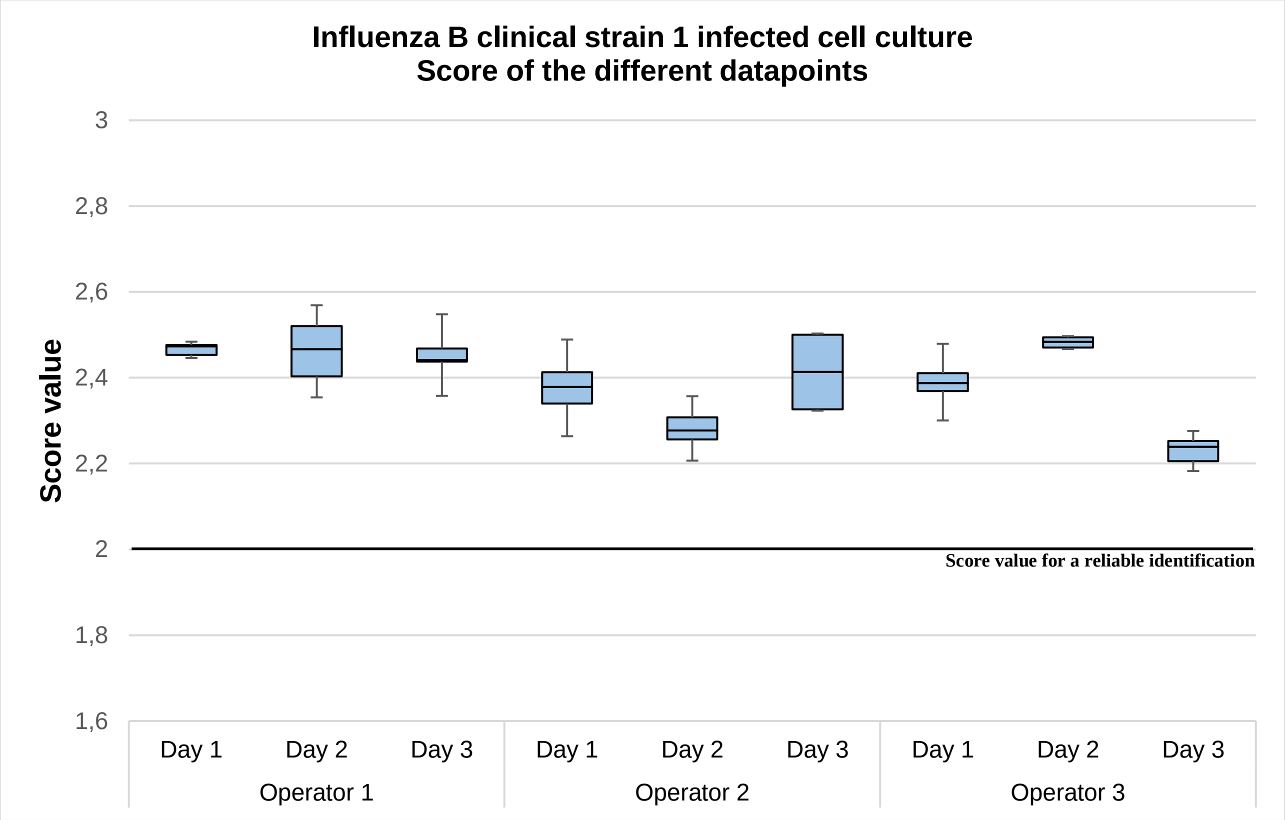


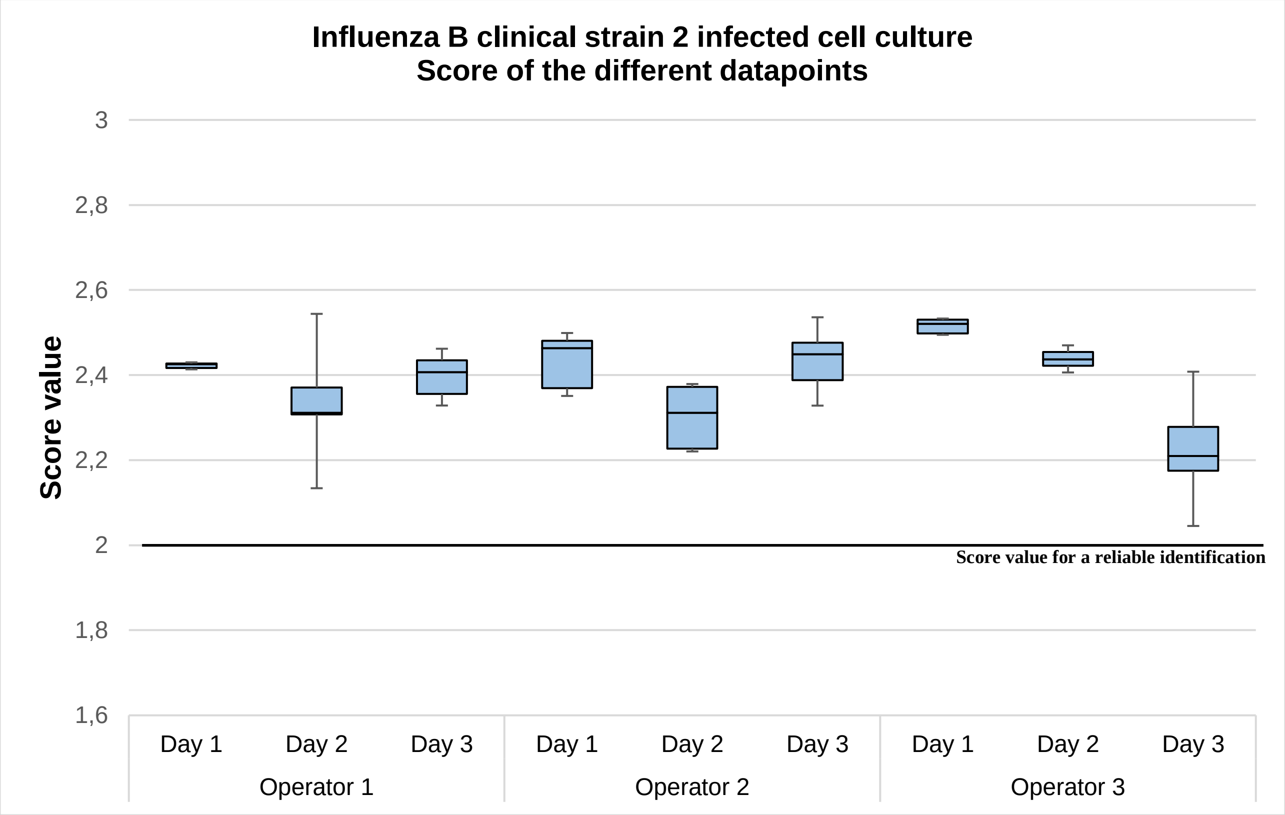


**Box plot showing the distribution of score values obtained for the replicates of 2 Influenza B clinical strains infected cell cultures by three independent operators in three different days.**

Box plot explanation: upper horizontal line of box, 75th percentile; lower horizontal line of box, 25th percentile; horizontal bar within box, median; upper horizontal bar outside box, 90th percentile; lower horizontal bar outside box, 10th percentile.

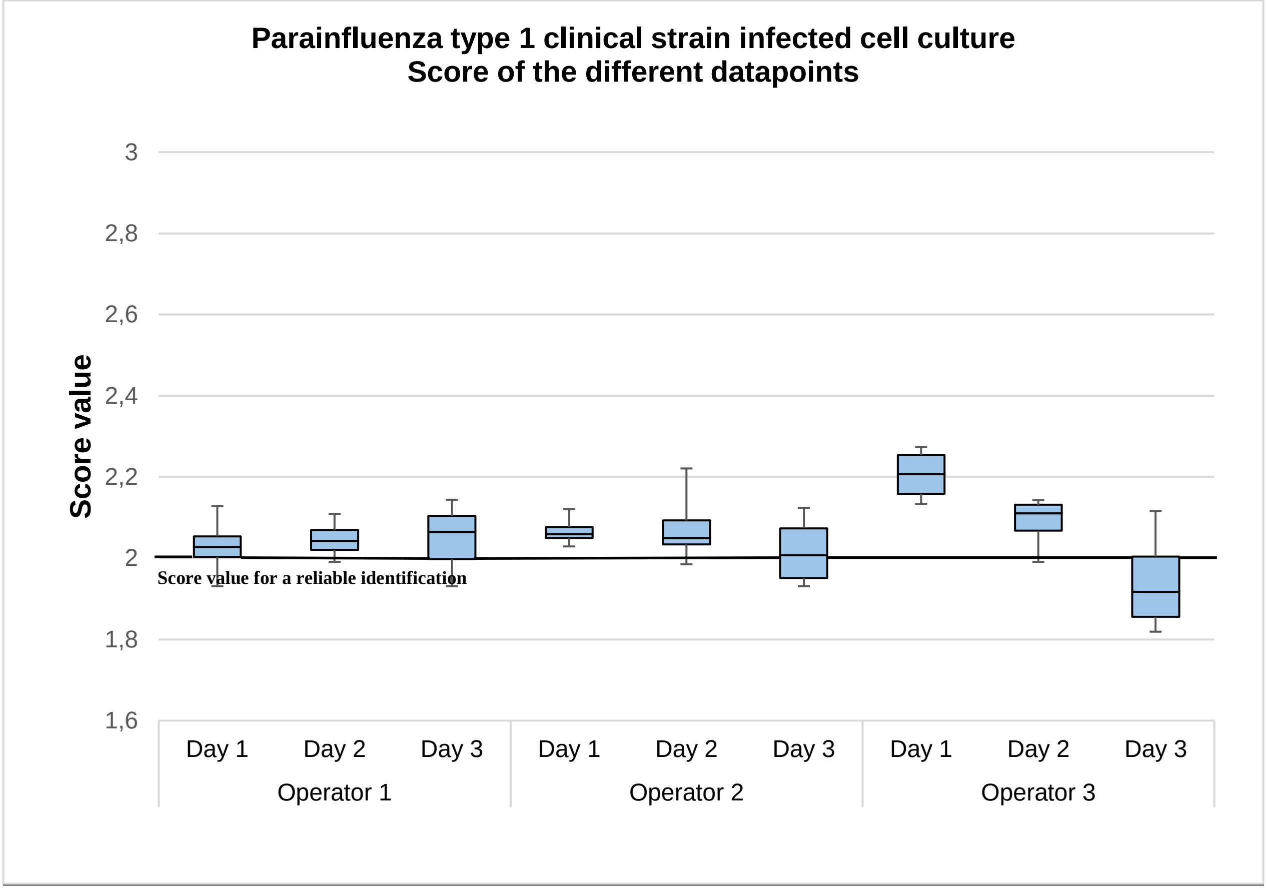


**Box plot showing the distribution of score values obtained for the replicates of Parainfluenza type 1 clinical strain infected cell culture by three independent operators in three different days.**

Box plot explanation: upper horizontal line of box, 75th percentile; lower horizontal line of box, 25th percentile; horizontal bar within box, median; upper horizontal bar outside box, 90th percentile; lower horizontal bar outside box, 10th percentile.

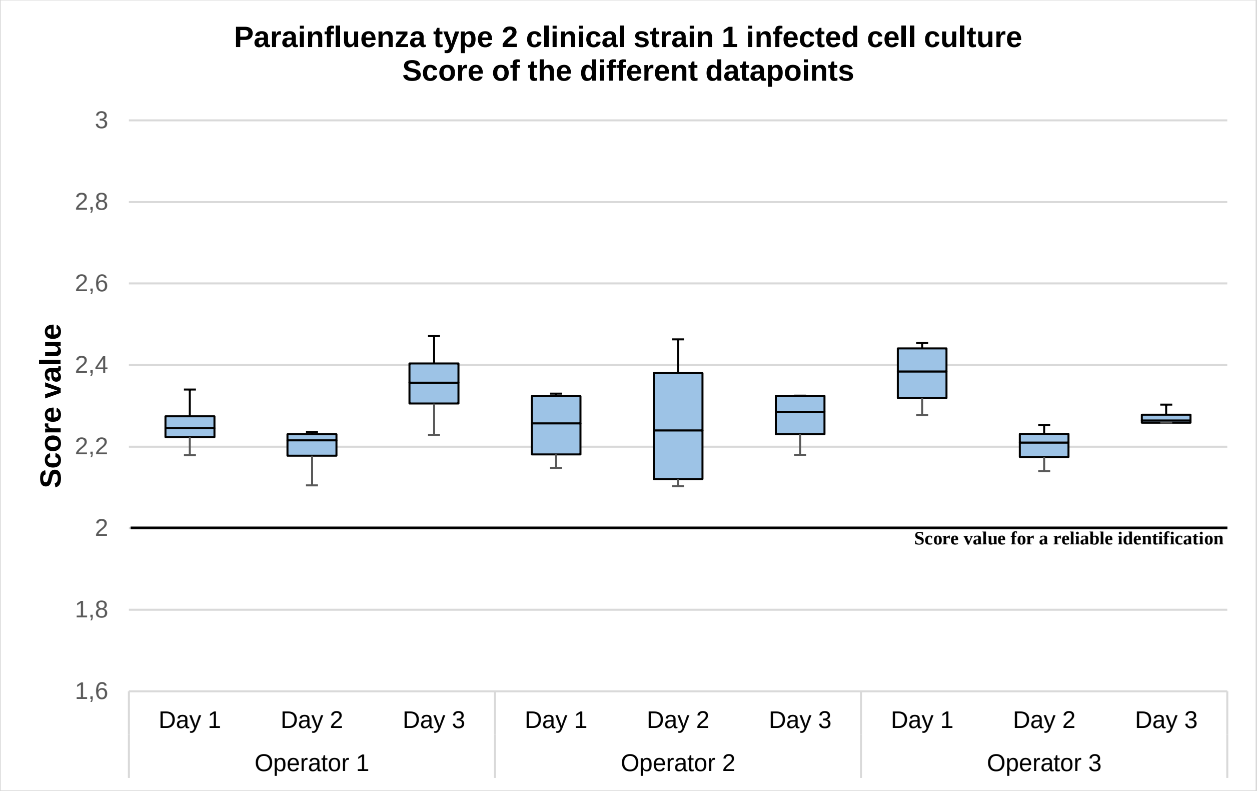


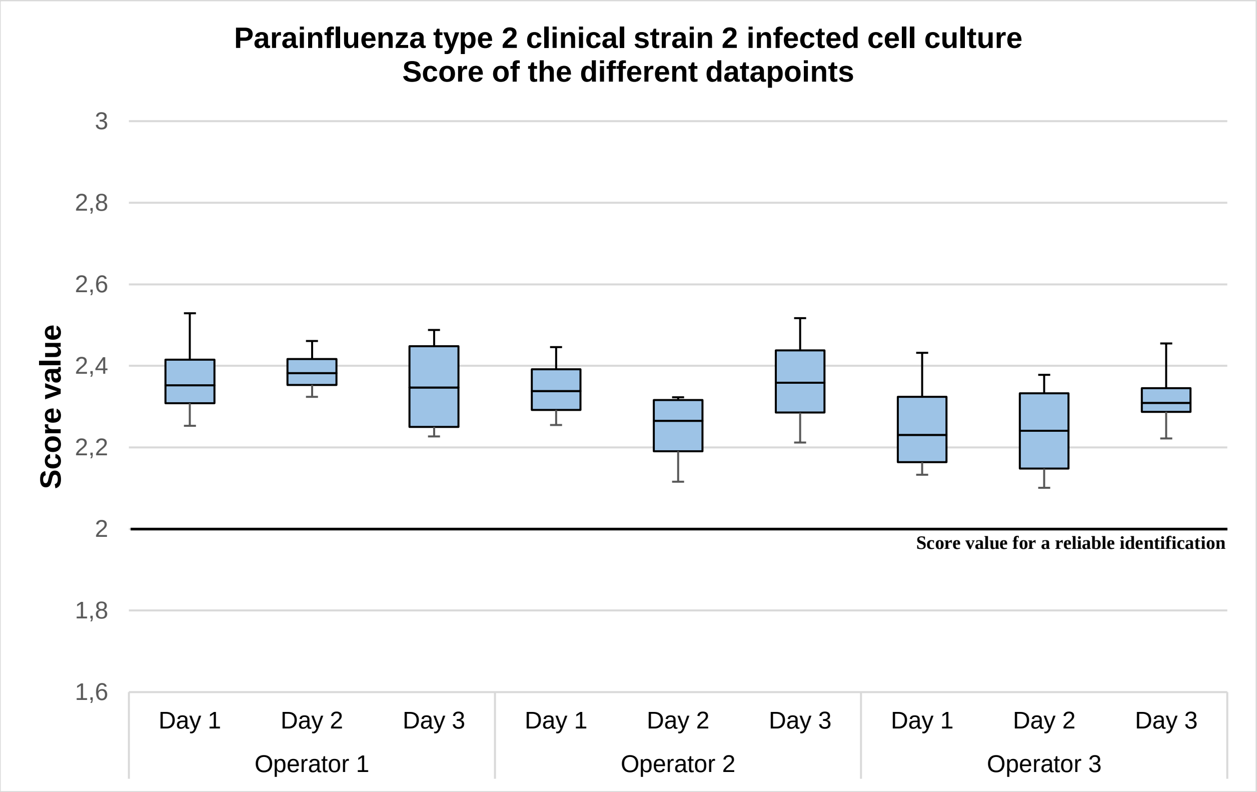


**Box plot showing the distribution of score values obtained for the replicates of 2 Parainfluenza type 2 clinical strains infected cell cultures by three independent operators in three different days.**

Box plot explanation: upper horizontal line of box, 75th percentile; lower horizontal line of box, 25th percentile; horizontal bar within box, median; upper horizontal bar outside box, 90th percentile; lower horizontal bar outside box, 10th percentile.

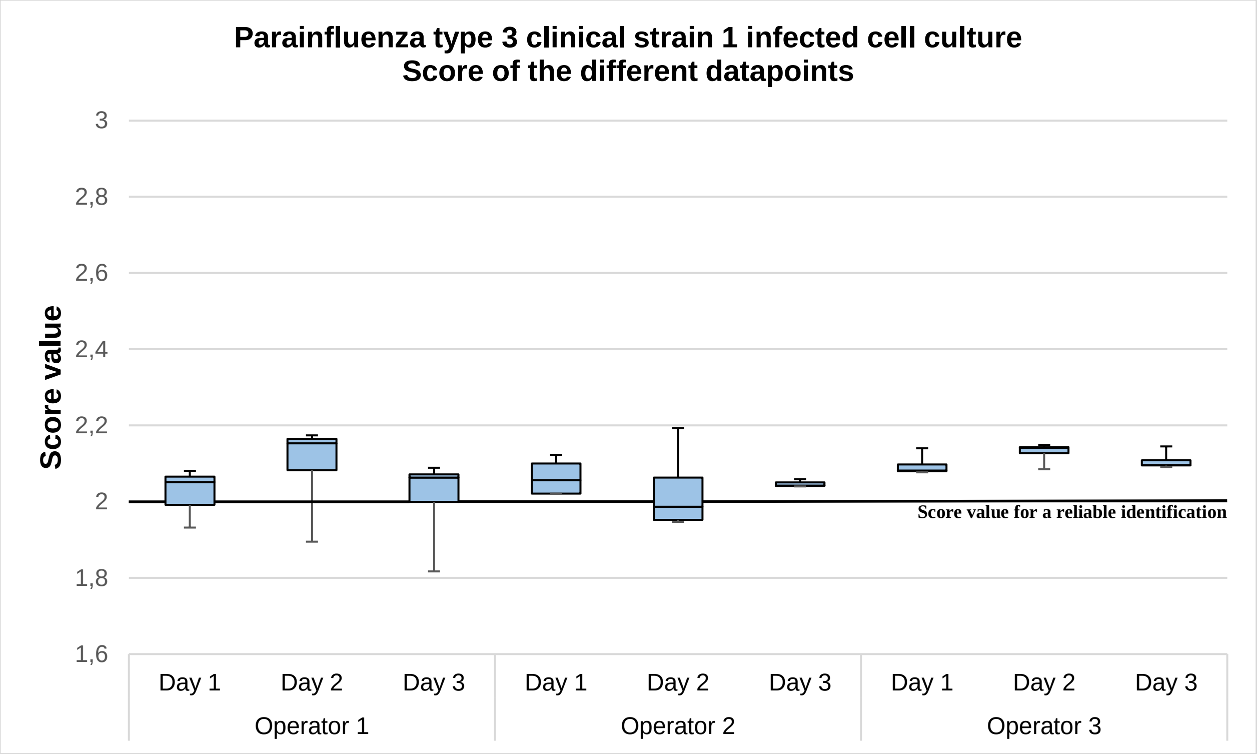


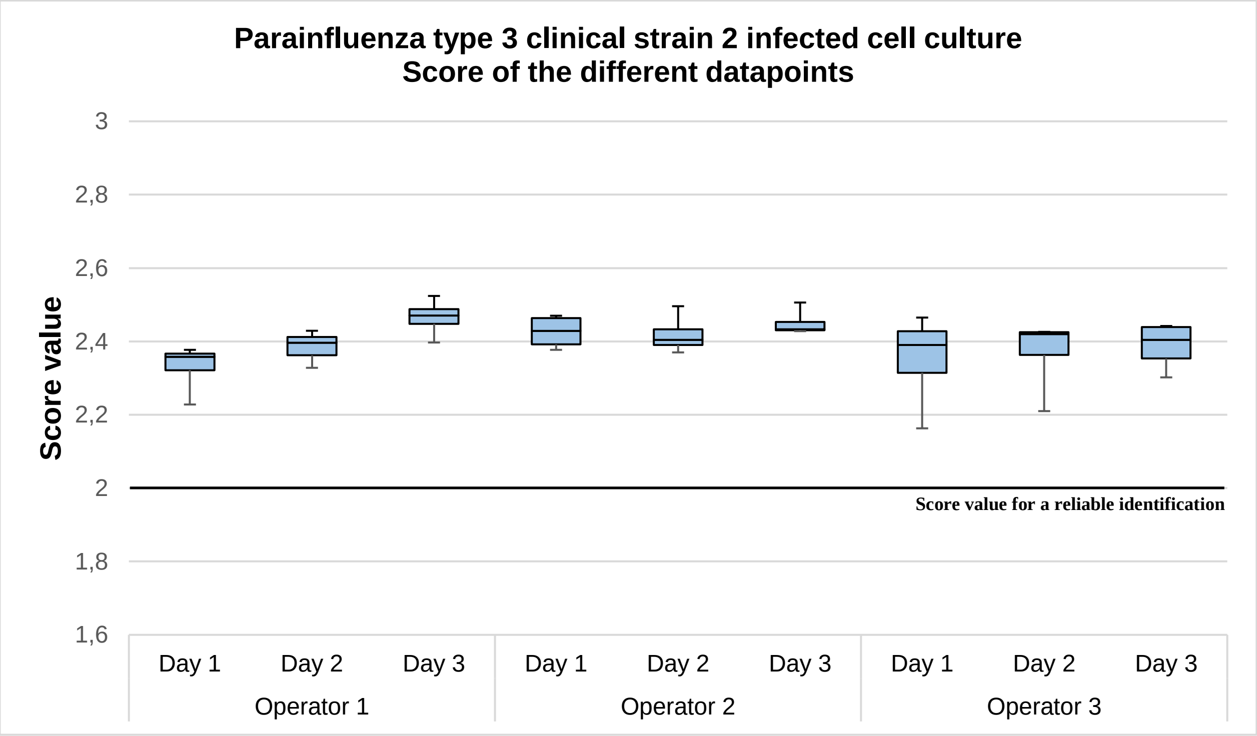


**Box plot showing the distribution of score values obtained for the replicates of 2 Parainfluenza type 3 clinical strains infected cell cultures by three independent operators in three different days.**

Box plot explanation: upper horizontal line of box, 75th percentile; lower horizontal line of box, 25th percentile; horizontal bar within box, median; upper horizontal bar outside box, 90th percentile; lower horizontal bar outside box, 10th percentile.

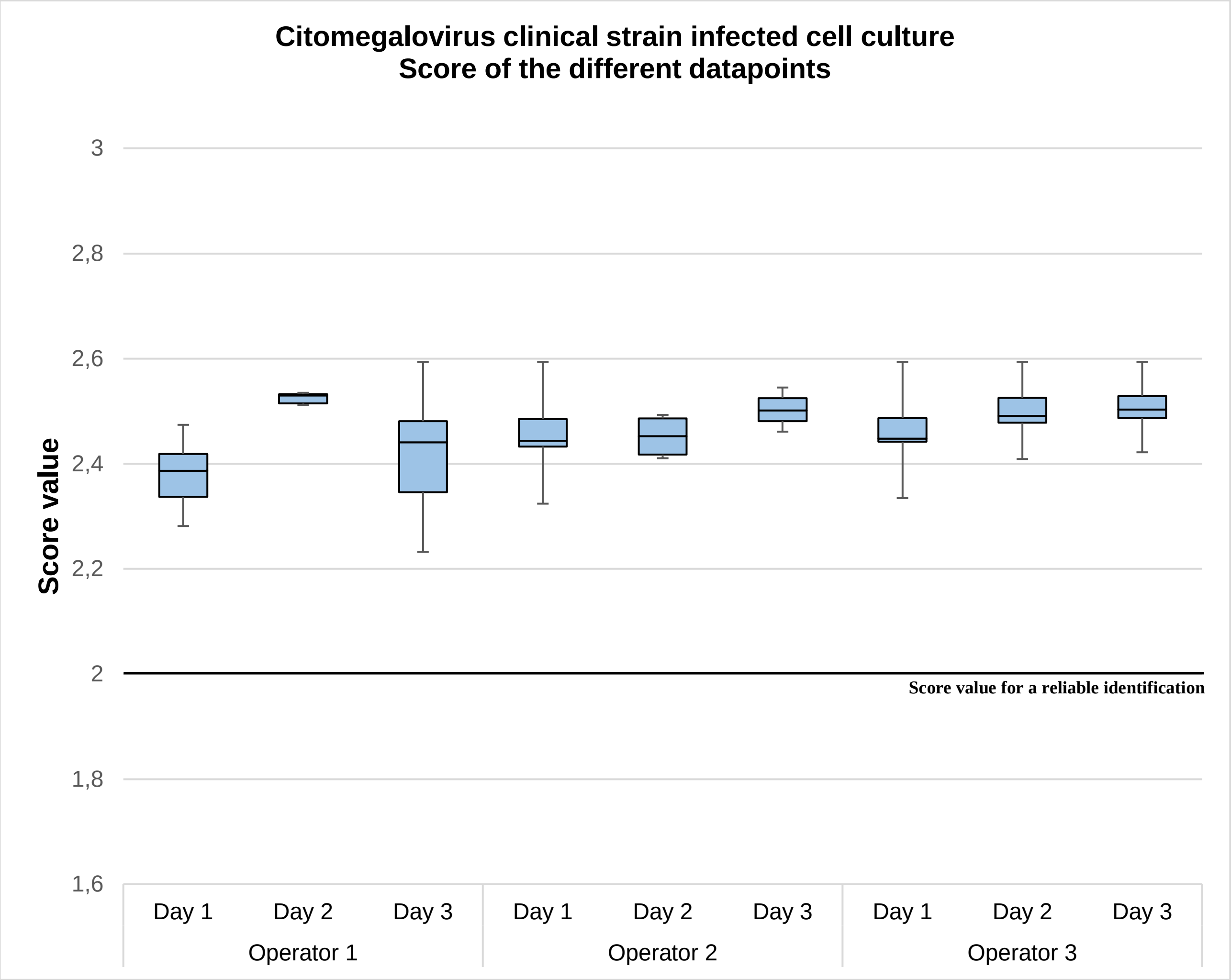


**Box plot showing the distribution of score values obtained for the replicates of Citomegalovirus clinical strain infected cell culture by three independent operators in three different days.**

Box plot explanation: upper horizontal line of box, 75th percentile; lower horizontal line of box, 25th percentile; horizontal bar within box, median; upper horizontal bar outside box, 90th percentile; lower horizontal bar outside box, 10th percentile.

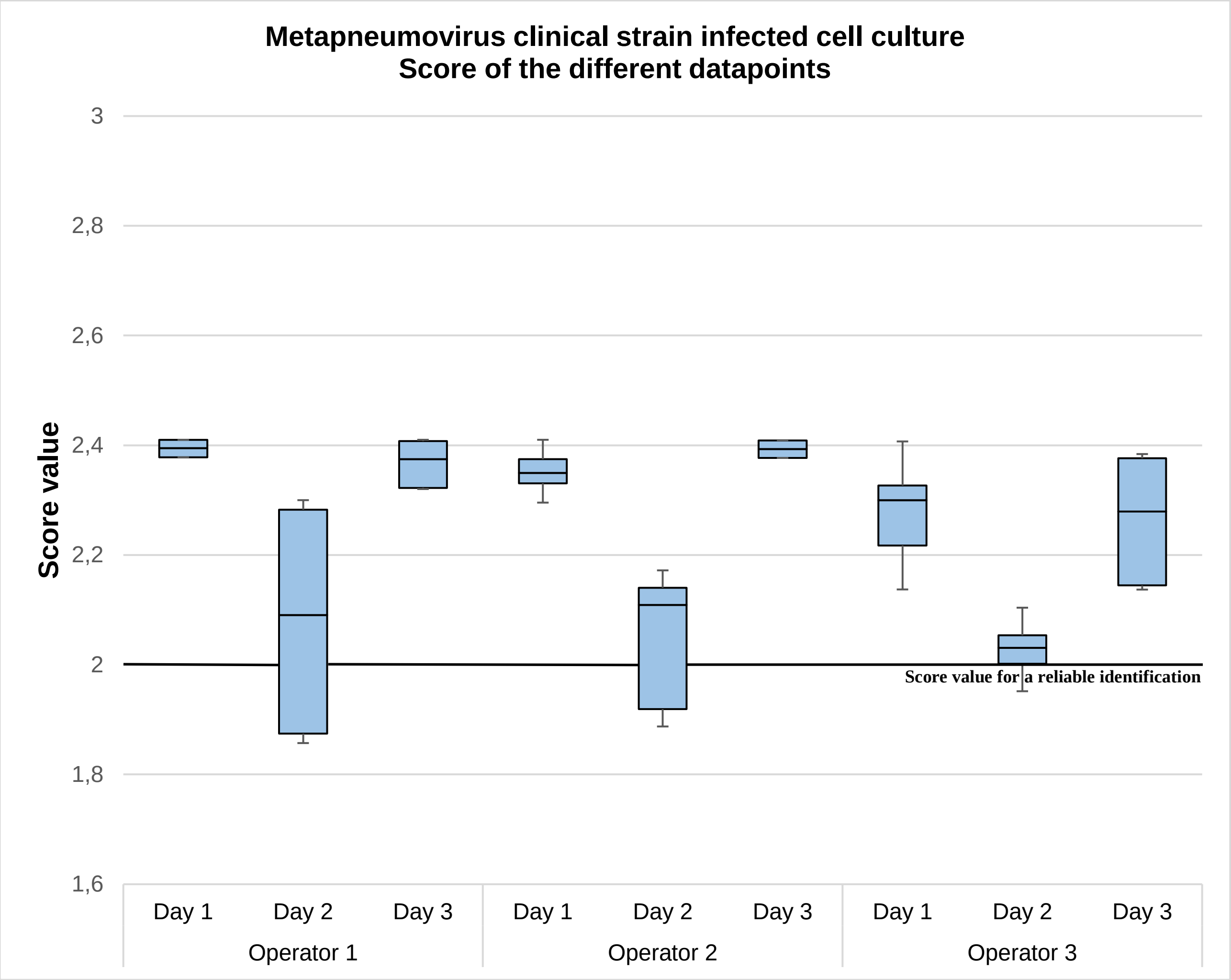


**Box plot showing the distribution of score values obtained for the replicates of metapneumovirus clinical strain infected cell culture by three independent operators in three different days.**

Box plot explanation: upper horizontal line of box, 75th percentile; lower horizontal line of box, 25th percentile; horizontal bar within box, median; upper horizontal bar outside box, 90th percentile; lower horizontal bar outside box, 10th percentile.
